# H_2_S Donors and Their Use in Medicinal Chemistry

**DOI:** 10.3390/biom11121899

**Published:** 2021-12-18

**Authors:** Elisa Magli, Elisa Perissutti, Vincenzo Santagada, Giuseppe Caliendo, Angela Corvino, Gianluca Esposito, Giovanna Esposito, Ferdinando Fiorino, Marco Migliaccio, Antonia Scognamiglio, Beatrice Severino, Rosa Sparaco, Francesco Frecentese

**Affiliations:** Department of Pharmacy, School of Medicine, University of Naples Federico II, Via D. Montesano 49, 80131 Napoli, Italy; elisa.magli@unina.it (E.M.); perissut@unina.it (E.P.); santagad@unina.it (V.S.); caliendo@unina.it (G.C.); angela.corvino@unina.it (A.C.); gianluca.esposito97@icloud.com (G.E.); giovannaesposito10@gmail.com (G.E.); fefiorin@unina.it (F.F.); marco.migliaccio94@libero.it (M.M.); antonia.scognamiglio@unina.it (A.S.); bseverin@unina.it (B.S.); rosa.sparaco@unina.it (R.S.)

**Keywords:** hydrogen sulfide, natural H_2_S donors, synthetic H_2_S donors, H_2_S release, medicinal chemistry

## Abstract

Hydrogen sulfide (H_2_S) is a ubiquitous gaseous signaling molecule that has an important role in many physiological and pathological processes in mammalian tissues, with the same importance as two others endogenous gasotransmitters such as NO (nitric oxide) and CO (carbon monoxide). Endogenous H_2_S is involved in a broad gamut of processes in mammalian tissues including inflammation, vascular tone, hypertension, gastric mucosal integrity, neuromodulation, and defense mechanisms against viral infections as well as SARS-CoV-2 infection. These results suggest that the modulation of H_2_S levels has a potential therapeutic value. Consequently, synthetic H_2_S-releasing agents represent not only important research tools, but also potent therapeutic agents. This review has been designed in order to summarize the currently available H_2_S donors; furthermore, herein we discuss their preparation, the H_2_S-releasing mechanisms, and their -biological applications.

## 1. Introduction

### 1.1. Hydrogen Sulfide: Chemical Properties

Hydrogen sulfide (H_2_S) is a colorless, flammable, and water-soluble gas well known for its characteristic odor of rotten eggs. The acute effects of H_2_S in humans are directly proportional to its concentration and these include eye, nose, throat, and respiratory system irritation (low concentrations) and rapid loss of consciousness and death (high concentrations) [1,2,3]. Recently H_2_S has been considered as a member of the gasotransmitter family because it shows some common characteristics with its congeners NO (nitric oxide) and CO (carbon monoxide) [4,5,6,7,8,9,10,11].

Hydrogen sulfide has a chemical structure similar to that of water, but it is much less polar than water because the sulfur atom is less electronegative than the oxygen present in water. For this reason, the intermolecular forces for H_2_S are relatively weaker and consequently the melting and boiling points are much lower than they are for water. H_2_S can be considered as a weak acid that gives rise to two dissociations in aqueous solution (Ka_1_ = 1.3 × 10^−7^ M, Ka_2_ = 1 × 10^−19^ M). From the first dissociation hydronium cation (H_3_O^+^) and hydrosulfide anion (HS^−^) are obtained; the second dissociation leads to H_3_O^+^ and sulfide ions (S^2−^). In biological fluids and under physiological conditions hydrogen sulfide is mainly undissociated [12].

Hydrogen sulfide represents a reductant species because of the oxidation state of the sulfur atom in H_2_S that is –2; it can also be considered a potent nucleophile at physiological pH values. These features make H_2_S a very reactive acid that can react with many biological molecules. Hydrogen sulfide can give rise to two kinds of redox reactions: the first oxidation should form a highly reactive HS^•^ radical (for example in the reaction with metal centers) [13], while the second kind of redox reaction of H_2_S with many biologically important reactive oxygen species (ROS) can also lead to the formation of species such as hydrogen peroxide (H_2_O_2_), superoxide (O_2_), peroxynitrite (ONOO), and NO. [14,15,16]. Hydrogen sulfide is also able to react with S-nitroso thiols to give thionitrous acid (HSNO) that probably acts as a cell-permeable nitrosylating agent that might represent the link and explain the cell membrane–impermeable transnitrosation mechanism [17]. Some evidence reported in the literature suggests that H_2_S also mediates an important oxidative post-translational modification and in particular protein S-sulfuration, generating, for example, S-SH groups on cysteine residues. Even if the detailed mechanism(s) is not yet known, this reaction could represent the chemical route involving H_2_S for the modification of the functions of a broad range of cellular proteins and enzymes. In the literature, authors have generally agreed that H_2_S reacts with cysteine-modified residues, such as disulfides (S-S–), sulfenic acids (S-OH), nitrosothiols (S-NO), etc., to form S-sulfhydrated products. Some authors have suggested that protein cysteines directly react with some H_2_S metabolites or precursors, such as sulfane sulfurs and hydrogen polysulfides, to obtain S-SH products [18,19,20,21,22,23,24,25,26,27]. However, further studies are needed to elucidate this mechanism in detail. Finally, H_2_S is able to bind transition metals by coordination bonds. In particular, it is able to bind strongly to copper, and this feature has allowed the development of fluorescent probes [28]. It has been shown that the activation of odorant receptors mediated by the sulfur–copper coordination leads to an increase in human sensitivity to the smell of volatile sulfur compounds [29].

The toxicity of hydrogen sulfide to mammals is due to the interactions with cytochrome c oxidase (CcO) with the consequent inhibition of mitochondrial respiration; however, the significant capacity of H_2_S to induce a suspended animation-like state [30] is believed to be associated with a reversible inhibition of CcO by sulfide coordination chemistry [31].

### 1.2. Hydrogen Sulfide: Biosynthesis and Metabolic Pathways

#### 1.2.1. Biosynthesis of H_2_S

**Figure 1:**
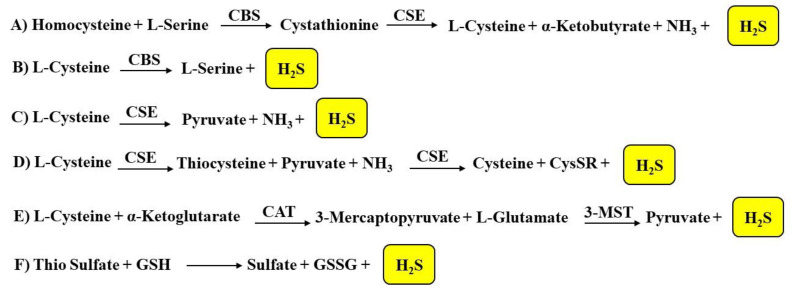
H_2_S biosynthesis: in the panels from (**A**–**F**) the different known pathways leading to the production of H_2_S in mammalian tissues are reported (CBS, cystathionine β-synthase; CSE, cystathionine γ-lyase; CAT, cysteine aminotransferase; MST, 3-mercaptopyruvate sulfurtransferase).

Hydrogen sulfide is produced by enzymatic and non-enzymatic endogenous reactions in mammalian tissues (Figure 1). The enzymatic production of H_2_S involves different enzymes such as cystathionine β-synthase (CBS) and cystathionine γ-lyase (CSE), that are two pyridoxal-5′-phosphate (PLP, vitamin B6)-dependent enzymes, as well as cysteine aminotransferase (CAT) and 3-mercaptopyruvate sulfurtransferase (3-MST). These enzymes interact with specific substrates and reverse trans-sulfuration pathways.

The human cystathionine beta-synthase (CBS) enzyme is made up of 551 amino acids and it is characterized by a subunit molecular weight of ~63 kDa [32]. CBS is a tetrameric protein, and each subunit binds to one heme and one pyridoxal 5′-phosphate (PLP). The catalytic center is similar to that of other members of the β- or fold II class of PLP-dependent enzymes and represents the conserved part of the protein [33]. Protoporphyrin IX represents a structural requirement of cystathionine β-synthase, and this feature is unique in the family of PLP-dependent enzymes. Moreover, a conserved glycine rich loop of fold II enzymes (G256–T257–G258–G259–T260) is responsible for multifarious electrostatic interactions with the phosphate portion of PLP [33]. 

The three-dimensional structures of human and yeast cystathionine gamma-lyase (CSE) have been determined through X-ray crystallography studies [34]. CSE is characterized by four equal monomers with a molecular weight of ~45 kDa; in each monomer the pyridoxal 5′-phosphate (PLP) cofactor is linked with a covalent bond. Nevertheless, some studies have reported differential PLP binding affinities within the monomers of CSE and a momentary dissociation or dismissal of PLP from the enzyme during the catalytic action [35]. Based on studies reported in the literature, several active site residues have been identified as being involved in the catalysis of the α,β-elimination reaction, leading to the production of H_2_S [36]. In the crystal structures of yeast and human CSE, some residues participate in the binding of the PLP cofactor. These residues are represented by Tyr^60^ and Arg^62^ from the adjacent monomer; Tyr^114^ that forms the π-stacking interaction with the pyridoxal moiety of the cofactor; Asp^187^ that establishes hydrogen bonds with the pyridoxal nitrogen; and Ser^209^ and Thr^211^ that form hydrogen bonds with the phosphate group. Some studies have also reported that Lys^212^ represents a significant catalytic residue considering that it forms a covalent bond with the PLP cofactor and facilitates proton transfer reactions during the α,γ-elimination reaction of L-cystathionine. Site-directed mutagenesis studies have demonstrated that the PLP cofactor also binds to Tyr^60^ and Arg^62^ from the adjacent subunit through a hydrogen bond [37].

CBS and CSE catalyze reactions that represent the primary pathways for H_2_S production (Figure 1). In particular, CBS is involved in the β-replacement reaction of homocysteine with serine to give cystathionine. CSE later takes part in the α,γ-elimination of cystathionine that leads to cysteine, α- ketobutyrate, NH_3_, and hydrogen sulfide (Figure 1A) [38]. 

Either CBS or CSE can also produce H_2_S from l-cysteine via β-elimination reactions [39]. In particular, CBS forms l-serine and H_2_S from l-cysteine (Figure 1B), while CSE generates pyruvate (using l-cysteine as the substrate), NH_3_ and H_2_S (Figure 1C) or it firstly forms thiocysteine, pyruvate, and NH_3_ and subsequently cysteine, S-alkenyl-mercaptocysteine (CysSR), and H_2_S (Figure 1D). Furthermore, CSE catalyzes the reaction of L-homoserine to form 2-oxobutanoate, NH_3_, and H_2_O.

CAT and 3-MST represent two other enzymes involved in the biosynthesis of hydrogen sulfide; CAT is able to transfer the amine group from cysteine to a keto acid (for example α-ketoglutarate) forming 3-mercaptopyruvate. Subsequently, 3-MST leads to the formation of the persulfide, 3-MST-SSH, through the desulfuration of 3-mercaptopyruvate (Figure 1E). 3-mercaptopyruvate sulfur-transferase then transfers the sulfur from 3-mercaptopyruvate to sulfite or other sulfur acceptors or forms elemental sulfur. The reaction is catalyzed by CAT and 3-MST and produces sulfane sulfur (or bound sulfur); subsequently, a reduction of the sulfur atom or a release from the thiosulfate or persulfides leads to the production of H_2_S. Therefore, the presence of reductants [40] and specific enzymes such as thiosulfate sulfur transferase or thiosulfate reductase is necessary to obtain hydrogen sulfide [41]. The enzymes involved in hydrogen sulfide biosynthesis are widely distributed. Both CBS and CSE represent cytosolic enzymes. CBS was initially thought to be predominantly involved in the production of H_2_S in the brain, while CSE was thought to be predominantly involved in the synthesis of H_2_S in the heart and blood vessels [42]. Subsequent studies have established a broader distribution of these enzymes and in particular, it was seen that CBS is present in vascular endothelium, CAT and 3-MST are present in the vascular endothelium and the brain, and 3-MST, but not CAT, is present in the vascular smooth muscle [43]. CAT and 3-MST constitute both mitochondrial and cytosolic enzymes [44], though 3-MST is believed to be predominantly present in the mitochondrion [45].

PGG (propargylglycine), BCA (β-cyanoalanine), AOAA (aminooxyacetic acid), trifluoroalanine, and HA (hydroxylamine) represent the most commonly used agents that inhibit H_2_S biosynthesis; in particular PGG and BCA can be considered specific CSE inhibitors, while AOAA can be considered as a CBS-selective inhibitor [7,46]. In particular, PGG is a specific CSE inhibitor in comparison to CBS, but it is able to interact with different PLP-dependent enzymes that are not necessarily involved in H_2_S production. In a study reported in the literature that monitored vascular disposition in cerebellar slices and in intact mouse brains and that foresaw the use of two-photon intravital laser scanning microscopy, Morikawa et al. analyzed the pathway mediating hypoxia-induced cerebral vasodilation. It was established that either H_2_S, obtained by cystathionine β-synthase (CBS), or CO, formed by heme oxygenase (HO)-2hypoxia caused cerebral vasodilation. Hypoxia reduces CO generation by HO-2, an oxygen sensor. The constitutive CO physiologically inhibits CBS, and hypoxia increases the H_2_S levels that leads to the vasodilation of precapillary arterioles. It has been observed that mice with a targeted deletion of HO-2 or CBS showed changed vascular responses to hypoxia. Therefore, the imaging mass spectrometry of an intact adult brain cerebral cortex of HO-2–null mice showed an impaired ability to preserve ATP levels during hypoxia [47].

As previously reported, H_2_S is also involved in non-enzymatic reactions (Figure 1F). In particular, the non-enzymatic reduction of elemental sulfur leads to a minor endogenous source of hydrogen sulfide; this reaction occurs using reducing equivalents obtained from the oxidation of glucose in erythrocytes [48]. Human erythrocytes are able to obtain H_2_S in the presence of elemental sulfur or inorganic polysulfides. Non-enzymatic oxidation sulfides provide thiosulfate and these can be converted to sulfite by enzymatic reactions with thiosulfate reductase in the liver, kidney, or brain, or by thiosulfate sulfur-transferase in the liver. H_2_S can also be released from thiosulfate and persulfides. Garlic-derived organic polysulfides have been proven to release hydrogen sulfide in a thiol-dependent manner [49]. Garlic (*Allium sativum*) has long been considered beneficial as an antioxidant; recent studies have demonstrated several favorable effects of garlic derived from H_2_S production. Allicin (diallylthiosulfinate) is the best characterized naturally occurring H_2_S-donating garlic compound; it decomposes in water to form compounds such as diallyldisulfide (DADS) and diallyltrisulfide (DATS).

#### 1.2.2. H_2_S Catabolic Pathways 

**Figure 2:**
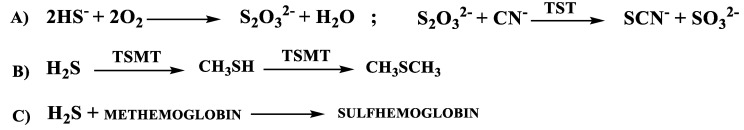
H_2_S catabolic pathways. (**A**) mitochondrial oxidation; (**B**) cytosolic methylation; (**C**) binding to hemoglobin. TST: thiosulfate cyanide sulfur transferase (rhodanese). TSMT: thiol-S-thiomethyl transferase.

The catabolism of hydrogen sulfide includes non-enzymatic and enzymatic reactions. In the first reaction of a catabolic pathway, H_2_S is rapidly oxidized, mainly in the mitochondria and in a non-enzymatic manner, to thiosulfate; subsequently, through an enzymatic reaction, thiosulfate is converted to sulfate and/or sulfite by TST (thiosulfate cyanide sulfur-transferase) (Figure 2A). Sulfite obtained by this reaction is then rapidly oxidized to sulfate; the latter is the major end-product of H_2_S catabolism under physiological conditions [50]. Another catabolic pathway of H_2_S is represented by the methylation to methanethiol and dimethyl sulfide mediated by TSMT (thiol-S-methyltransferase) (Figure 2B). This latter catabolic pathway occurs in the cytosol of different cells of the gastrointestinal tract [51,52]. The last catabolic reaction of H_2_S involves the methemoglobin that is able to bind to endogenous gases (for example CO and NO); moreover, it is able to bind H_2_S to give sulfhemoglobin (Figure 2C) [53].

The catabolism of H_2_S in the mitochondria is related to the coupled activity of several mitochondrial enzymes (SQR, ETHE1, SO, of which the initial one (SQR) is oxygen-independent) and their substrates (CoC, GSH glutathione, O_2_). In order to form thiosulfate, H_2_S is initially processed by SQR and GSH to form glutathione persulfide (GSSH) that serves as a substrate for both sulfite and thiosulfate formation. Alternatively, H_2_S can be processed by SQR and sulfite to form thiosulfate. Most of the catabolic reactions regarding H_2_S in mitochondria are enzymatic [17].

### 1.3. Hydrogen Sulfide: Biological Functions

The scientific literature concerning the biological roles for endogenous hydrogen sulfide is continuously growing. Several studies have reported that H_2_S performs physiological effects at a broad range of concentrations (10–300 μM) [5,6,7]. It is known that H_2_S participates in the regulation of numerous physiological responses, for example, anti-inflammation [54], oxidative stress [55], neuromodulation [56], vasoregulation [57], protection from reperfusion injury after myocardial infarction [58], and insulin resistance [59]. Furthermore, scientific research has continued to investigate H_2_S in relation to its involvement in various phases of cellular signaling, cell function, and cytoprotection.

#### 1.3.1. Cardiovascular System

As reported in several studies, H_2_S has biological significance in the control of cardiovascular homeostasis. Particularly, it has been shown that H_2_S presents all the positive effects of NO without forming toxic metabolites [60]. Lately, it has been observed that the function of H_2_S in cardiovascular homeostasis is more important and crucial in cases of NO-mediated control compromise such as in the endothelial dysfunction. Hydrogen sulfide induces relaxing effects in the vascular smooth muscle and this effect has been observed in large vessels, for example, the rat thoracic aorta and portal vein and also in peripheral resistance vessels, which have a more substantial role than large conduit arteries in the modulation of vascular resistance and blood pressure [61]. l-cysteine represents a source of H_2_S; in fact, the vasorelaxing activity of l-cysteine is reduced by the CSE inhibitor PGG. Genetic deletion of CSE in mice significantly decreases H_2_S levels in the serum, heart, and aorta, and mutant mice lacking CSE presented with marked hypertension and a reduced endothelium-dependent vasorelaxant effect [62]. In fact, it is known that H_2_S relaxes blood vessels primarily, but not exclusively, by opening the K_ATP_ channels of the vascular smooth muscle cells. In addition to the vasorelaxing effect, H_2_S, as well as NO, is characterized by a broad range of other biological effects, which are significant for the polyhedric control of the cardiovascular system. For example, H_2_S is able to inhibit the platelet aggregation/adhesion induced by ADP, collagen, epinephrine, arachidonic acid, thromboxane mimetic U46619, and thrombin [63].

#### 1.3.2. Immune System and Inflammation

The production and functional role of H_2_S is important in the several cell types classically related to innate immunity (such as neutrophils, eosinophils, basophils macrophages, dendritic cells, natural killer cells, mast cells,) and adaptive immunity (T and B lymphocytes). H_2_S is also associated with the progress of various inflammatory and immune diseases. In this context, H_2_S has physiological and pathophysiological roles especially in the oral cavity and in the colon, where endogenous bacteria produce H_2_S giving rise to a “H_2_S environment” to which the immune cells and the parenchymal cells are subjected; this “H_2_S environment” significantly regulates their viability and function. The development of spontaneous autoimmune disease, the onset, or the worsening of the severity of various immune-mediated diseases (such as autoimmune rheumatoid arthritis or asthma) could be connected to the downregulation of endogenous H_2_S-producing enzymes, or to the genetic defects in H_2_S biosynthetic enzyme systems. Small amounts of H_2_S, delivered by donors, enhance the function of various immune cells, and defend them against the dysfunctions caused by various noxious stimuli. The effects of H_2_S preserve the immune functions, increase the antimicrobial defenses, and exercise anti-inflammatory therapeutic effects in various diseases. 

As is already known, nonsteroidal anti-inflammatory drugs (NSAIDs) are able to induce gastroenteropathy [64]. Studies reported in the scientific literature suggest that NSAIDs repress endogenous H_2_S synthesis by decreasing the expression of CSE. The additional decrease in H_2_S synthesis may sequentially cause an expansion in leukocyte adherence leading to the development of the gastric injury that has been noted subsequent to NSAID administration [65]. Equally, the administration of exogenous H_2_S decreases the capacity of these agents to affect gastric injury. Exogenous administration of H_2_S repressed NSAID-induced granulocyte infiltration, the expression of endothelial and leukocyte adhesion molecules, and the expression of TNFα (tumor necrosis factor α) [64]. It has been shown that leukocyte adhesion to the vascular endothelium produced by aspirin injury decreases after improvements in H_2_S bioavailability and that CSE inhibition with propargylglycine (PGG) impaired aspirin-mediated mucosal injury and inflammation. It has been also observed that the leukocyte expression of LFA-1 is repressed by exogenous H_2_S. It is also important to highlight a molecular characteristic of the H_2_S-induced anti-inflammatory effects: H_2_S donors reduced aspirin-induced leukocyte adhesion as a result of the activation of K_ATP_ channels and the inhibition of the CSE activity that encourages leukocyte adhesion. Further studies have shown that the co-administration of an H_2_S donor with an NSAID leads to the inhibition of NSAID-induced leukocyte adherence and a decrease in the gravity of gastric injury [66].

#### 1.3.3. Respiratory System

The relaxant effects of H_2_S have also been explored on the rings of the bronchial smooth muscle of two rodent species. Fu et al. demonstrated that H_2_S produced a strong relaxation in the isolated bronchus rings from the mouse but generated only minor relaxation in guinea pig rings. H_2_S content and CSE activity were also improved in an isolated rat lung which presented with an ischemia/reperfusion injury, and a preventative perfusion with H_2_S reduced this injury, decreasing malondyaldehyde (MDA) production and increasing superoxide dismutase and catalase activity [67]. 

**Figure 3:**
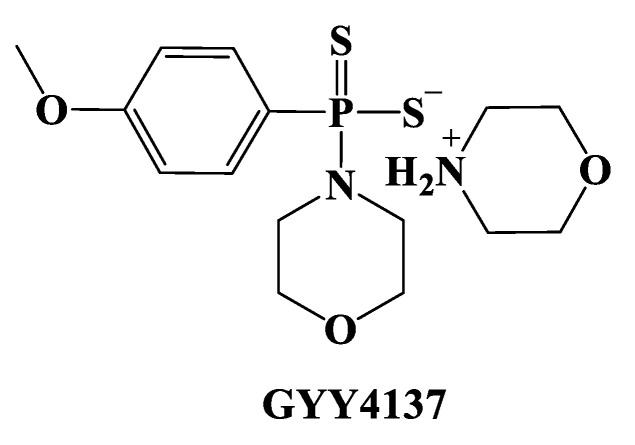
Chemical structure of GYY4137.

The endogenous CSE/H_2_S pathway seems to have an important role in pulmonary hypertension (PH). Specifically, this pathway was down-regulated in hypoxic PH (HPH), causing a reduction in endogenous H_2_S production in rat lung tissues as a result of oxidative stress. In lung tissues, the gene expression, and the activity of the CSE enzyme were suppressed during HPH, but the exogenous amount of H_2_S led to an improvement in CSE activity, an upregulation of CSE gene expression in the lung tissue, and a decrease in the remodeling of the pulmonary vascular structure during HPH [68]. Subsequently, after the treatment with exogenous H_2_S, HPH was reduced because of a direct scavenging of oxidized glutathione (GSSG), an improved total antioxidant capacity [69], and the inhibitory effect of H_2_S on pulmonary vascular inflammation, connected to a high I-B (inhibitor of NF-B) expression and a down-regulation of NF-B p65 expression [70]. Moreover, the CSE/H_2_S pathway seems to also have a favorable effect in asthma and chronic obstructive pulmonary disease (COPD), two of the most important obstructive airway diseases. In milder COPD, enhanced levels of H_2_S serum may have a beneficial effect in airway protection, antagonizing oxidative stress and airway inflammation and blocking the progress of COPD [71]. Scientific research has shown many positive effects for H_2_S in ischemia for several tissues. H_2_S has a broad range of protective functions related to ischemia/reperfusion (I/R) injury all through the body. The control of intracellular [Ca^2+^] through the stimulation of the KATP channel opening, anti-apoptotic pathway (ERK1/2/MAPK, PI-3 kinase/Akt, JAK-STAT) activation, mitochondrial protection through the preservation of ΔΨm and the inhibition of the mPTP opening, and the anti-inflammatory effects through the activation of eNOS and p38 MAPK represent the mechanisms of protection [72]. H_2_S has been also revealed to be protective against pulmonary ischemia injury and the endogenous CSE/H_2_S pathway could be implicated in the pathogenesis of lung I/R injury [67]. In a recent study, Jiang et al. showed that diabetes mellitus (DM) exacerbated lung I/R injury and that oxidative stress had a key role in this process. It was revealed that H_2_S had a protective effect against diabetic lung I/R injury by silencing oxidative injury. In this study, the mechanism by which H_2_S affected diabetic lung I/R injury was examined. Streptozotocin-induced type 2 diabetic rats fed a high-fat diet were exposed to GYY4137 (Figure 3), a slow-releasing H_2_S donor in the presence of and in the absence of EX527 (a SIRT1 inhibitor), and later exposed a surgical model of IR injury of the lung. Subsequently, the lung function, oxidative stress, cell apoptosis, and inflammation were assessed. It was observed that the damage to the lung SIRT1 signaling under type 2 diabetic conditions was additionally intensified by I/R injury. GYY4137 treatment significantly stimulated SIRT1 signaling and enhanced the I/R injury in the lung in type 2 DM animals through an improvement in the functional recovery of the lung, reducing oxidative damage, reducing inflammation, and destroying cell apoptosis. Nevertheless, these effects were mainly compromised by EX527. Furthermore, treatment with GYY4137 substantially stimulated the Nrf2/HO-1 antioxidant signaling pathway and improved eNOS phosphorylation; these effects were largely eliminated by EX527. These results suggest that GYY4137 treatment significantly reduced lung I/R injury under type 2 diabetic conditions via the activation of lung SIRT1 signaling. SIRT1 activation upregulated Nrf2/HO-1 and activated the eNOS-mediated antioxidant signaling pathway, consequently decreasing cell apoptosis and inflammation, and finally maintaining+ lung function [73].

#### 1.3.4. Central Nervous System

H_2_S exerts important regulatory effects in the control of important functions in the Central Nervous System (CNS). A high expression of CBS in the rat hippocampus and cerebellum has been observed [56]. H_2_S improves cAMP production, giving an enhanced sensitivity towards NMDA receptors to glutamate in CNS neurons [74]. This sensitization of the NMDA receptors, stimulated by concentrations of H_2_S between 50 and 160 μM, promotes the induction of hippocampal long-term potentiation, a process of synaptic plasticity implicated in the mechanisms of learning and memory [75]. The improved production of cAMP stimulates protein kinase A which controls brain function by intracellular protein phosphorylation; moreover, H_2_S may increase the reducing activity and defend neurons against oxidative stress through the activation of upstream receptor tyrosine kinase [76]. Lastly, some studies have reported that H_2_S reduces lipopolysaccharide (LPS)-induced NO production in the microglia through the inhibition of p38-MAPK [77] and that MAPKs regulate cellular activities [78]. The obtained data indicate an important role for H_2_S in the treatment of cerebral ischemia and neuro-inflammatory diseases.

#### 1.3.5. Reproductive System

Studies in the scientific literature have reported several effects of exogenous H_2_S on mammalian reproductive systems. H_2_S has an important physiological role in the erectile response of the human corpus cavernosum due to the vasorelaxant effect on the penile artery. D’Emmanuele di Villa Bianca et al. demonstrated that l-Cys or NaHS produces a relaxation of the human corpus cavernosum strips in a concentration-dependent manner. l-Cys relaxation was inhibited by AOAA (aminoxyacetic acid; CBS inhibitor). It was shown that PAG (propargylglycine; CSE inhibitor) or AOAA potentiated the electrical field stimulation of human penile tissue. In rats, l-Cys and NaHS were shown to promote penile erection and the response to l-Cys was impeded by PAG. The acquired data suggest that the l-Cys/H_2_S pathway facilitates human corpus cavernosum smooth-muscle relaxation [79].

#### 1.3.6. Gastrointestinal System

Several papers concerning the role of H_2_S in the gastrointestinal (GI) tract have been described in the scientific literature. Studies on H_2_S-induced chloride secretion proved that H_2_S is able to stimulate primary afferent nerve fibers in the mucosa/submucosa of the colon in guinea pigs and humans and this leads to increased chloride secretion. Hydrogen sulfide determines the contractile function either acting directly on the smooth muscle itself or by stimulating neurons in the enteric nervous system that influence smooth muscle function. Even if the effect of H_2_S on inflammation is controversial and many studies have demonstrated its anti-inflammatory effect at physiological concentrations, H_2_S can be considered as a proinflammatory mediator in abdominal sepsis, endotoxemia, and pancreatitis counter to its anti-inflammatory effects in animal models of gastritis and colitis [80,81,82].

In recent years, the role of H_2_S and other gaseous mediators (such as CO and NO) in digestive systems has been highlighted. The cytoprotective and anti-inflammatory properties of H_2_S are correlated to mucosal defense and ulcer healing in the GI system [83]. H_2_S is also involved in the physiology of the intestinal microbiota and in particular, it is able to inhibit pathogenic bacteria and to promote mucus production [84]. Furthermore, H_2_S-releasing derivatives of non-steroidal anti-inflammatory drugs (HS–NSAIDs) have been proven to reduce the gastric damage in comparison to the corresponding parent drugs [85]. H_2_S donors have also been shown to alleviate visceral pain and HS–trimebutine has been tested in clinical trials as an abdominal analgesic (NCT01926444) [86,87,88].

### 1.4. Hydrogen Sulfide and Diseases

Hydrogen sulfide is involved in several physiological and pathophysiological processes; this endogenous mediator is in fact associated with the homeostasis of the cardiovascular, renal, and central nervous systems and to dermatological diseases, various types of cancer, and viral infections as well as the SARS-CoV-2 infection that leads to COVID-19.

Hypertension, atherosclerosis, hyperhomocysteinemia, and diabetes represent cardiovascular diseases that are connected to vascular endothelial dysfunction; research has shown that H_2_S can be considered as a vasoprotective gasotransmitter in diseases where endothelial dysfunction is the central issue [89]. Moreover, morphological and functional modifications at the endothelial level correspond to the first events of cell senescence that inexorably result in vascular dysfunction and the subsequent atherosclerosis that profoundly impact cardiovascular health. In fact, myocardial hypertrophy and fibrosis can certainly arise and can lead to a compromise in the general cardiac output. Interestingly, hydrogen sulfide has been recently identified as a gaseous molecule able to manipulate the intracellular pathways implicated in cell senescence and so it is considered as a possible target for blocking cardiovascular diseases. In particular, the correlation between the decrease in endogenous H_2_S levels and the beginning of several cardiovascular cell senescence-related diseases has been demonstrated [90].

Numerous studies have clearly demonstrated that H_2_S has a considerable protective effect in myocardial ischemia. The processes for H_2_S cardioprotection involve the regulation of ion channels; antifibrotic and antiapoptotic effects; a reduction in oxidative stress and inflammatory response; the protection of mitochondria; the regulation of microRNA expression; and the promotion of angiogenesis. H_2_S donors could be used to develop effective drugs for the care of cardiovascular diseases such as myocardial ischemia [91,92].

Hydrogen sulfide has a crucial role in blood pressure control and renal physiology too. In the kidney, H_2_S is a significant regulator of vascular and cellular function, although the processes that influence the (sub)cellular levels of H_2_S are not exactly understood. H_2_S regulates systemic and renal blood flow, the amount of glomerular filtration, and the renin–angiotensin axis by direct inhibition of nitric oxide synthesis. In addition, H_2_S affects cellular events by altering protein activity through post-translational protein modification. This process represents persulfidation and is able to modulate protein localization, protein activity, and protein–protein interactions. Furthermore, acute kidney injury (AKI) caused by mitochondrial dysfunction, which happens in the course of hypoxia or ischemia-reperfusion (IR), is mitigated by H_2_S. H_2_S increases ATP production, inhibits the injury caused by free radicals, and controls endoplasmic reticulum stress during IR [93]. Therefore, forerunners for H_2_S endogenous synthesis, H_2_S donors, and natural plant-derivative compounds may serve as strategies to prevent hypertension and kidney disease [94]. Corvino et al. recently reported a survey of currently available H_2_S donors and their effects on cardiovascular disease; these H_2_S donors have been described as the basis of the mechanism that triggers the release of H_2_S and underline their possible use as favorable drugs in the treatment of cardiovascular diseases [95].

H_2_S, NO, and their crosstalk, have been described as helpful in Acute Kidney Injury (AKI), a syndrome mainly produced by ischemia and reperfusion (IR) injury, which momentarily impedes the blood flow, improves inflammation processes, and stimulates oxidative stress. Both these endogenous gasotransmitters act by reducing inflammation, regulating reactive oxygen species (ROS) concentrations, and modulating pro-inflammatory cytokines, as well supporting vasodilation and reducing hypertrophy, apoptosis, and autophagy [96].

Some studies reported in the scientific literature describe the correlation between H_2_S and cisplatin-induced nephrotoxicity. Precisely, the decrease in H_2_S by cystathionine γ-lyase (CSE) downregulation, subsequent to cisplatin treatment, may contribute to cisplatin-induced renal cell injury, probably through the increase in the production of endogenous reactive oxygen species (ROS); on the other hand, H_2_S prevents successive renal disorders through the inhibition of the activation of NADPH oxidase. Interestingly, GYY-4137, a slow-releasing H_2_S donor, has been proven to improve the anticancer activity of cisplatin in various cancer cell lines, maybe because of its specific anticancer effect. Nevertheless, the effectiveness of H_2_S donors in tumor-bearing animals continues to be examined in terms of renal protection, cancer inhibition, and later, cisplatin treatment. Moreover, more data regarding the use of polysulfide, a H_2_S derived compound, in the therapy of cisplatin-induced nephrotoxicity, have been additionally reported [97].

During recent years, a wide range of studies have focused on the function of H_2_S in the central nervous system (CNS), providing evidence that H_2_S has an important role in neuroprotection activities because it constitutes a signaling molecule that controls the function of the CNS. Neurodegenerative diseases are linked to endoplasmic reticulum stress (ERS) and protein misfolding. H_2_S regulates ERS and shows a broad variety of cytoprotective and physiological functions in CNS degenerative diseases. The neuroprotective effect of H_2_S for ERS has an important role in various CNS diseases as well as Parkinson’s disease, depression disorders, and Alzheimer’s disease [98].

In bone tissue, H_2_S has a cytoprotective role and stimulates bone formation; recently, scientific studies have established its role as a therapeutic agent in bone pathologies. H_2_S has been proven to stimulate several signaling pathways implicated in several phases of the processes of bone repair. In particular, H_2_S seems to be able to modulate osteogenic differentiation in osteoprogenitor cells through the induction of S-sulfhydration in TRP channels, increasing Ca^2+^ influx in the mesenchymal stromal cells (MSCs); H_2_S donors encourage the expression of osteogenic signaling by the stimulation of multiple signaling pathways at the transcriptional level. Concerning inflammation, hydrogen sulfide activates antioxidant signaling in osteoclasts (OCs), which includes the improved nuclear translocations of NRF-2 and leads to the inhibition of OC differentiation. Pharmacological administration of H_2_S has led to promising outcomes in preclinical studies in the treatment of systemic bone diseases, for example, osteoporosis; nevertheless, local delivery of H_2_S offers further opportunities for treatment. Moreover, new H_2_S donors and biomaterials have recently been studied, paving the way for the advancement of H_2_S-releasing procedures for bone re-forming medicine [99].

Alterations in H_2_S production are involved in several dermatological diseases, for example, psoriasis, melanoma, and other dermatoses. The consequences of H_2_S in the skin involve the stimulation of keratinocyte differentiation; the control of vasodilatation; wound healing; the regulation of apoptosis; the modulation of pruritus; and the resolution of inflammation. Regarding the H_2_S pathway in the skin, the information available references the connection between this molecule and the physiological and pathological effects. Generally, most of the effects of H_2_S are clearly concentration or dose-dependent. Due to the important role of H_2_S in skin physiology, it is not unexpected that modified H_2_S production leads to the pathogenesis of several cutaneous diseases. Many H_2_S donors have been used in experiments, and different results have been acquired. Nevertheless, these compounds present different intrinsic characteristics such as the capacity to enter the cell or organelle specifically, H_2_S-releasing kinetics, and pH dependence, but certainly, these chemical entities include a new family of potential therapeutic tools for the treatment of various skin diseases by targeting their differential etiological aspects. Obviously, the development of H_2_S donors will necessarily rely on an better understanding of the exact role of H_2_S and its goals in the various phases of skin biology [100].

Several studies reported in the scientific literature have shown an effective role of H_2_S in the framework of cancer biology. CSE, CBS, and 3MST, the three H_2_S-producing enzymes, are expressed in several types of cancer. Furthermore, the inhibition of CBS leads to anti-tumor activity especially in breast cancer, ovarian cancer, and colon cancer, while the effect of CSE or 3-MST inhibition has been largely unexplored in cancer cells. Interestingly, H_2_S donation has been shown to induce cancer cell apoptosis in in vitro and in vivo studies. In particular, low levels of H_2_S may show pro-cancer effects, while higher levels of H_2_S may lead to cancer cell death. This leads us to assume that the inhibition of H_2_S biosynthesis and H_2_S supplementation work in two different ways in relation to cancer treatment. This enigmatic role of H_2_S is interesting in relation to the development of innovative CBS inhibitors, H_2_S donors, and H_2_S-releasing hybrids that could therefore have potential for cancer therapy [101].

Finally, it is possible to affirm that hydrogen sulfide represents a natural defense against the infections from enveloped RNA viruses and is implicated in COVID-19. It has previously been reported that H_2_S inhibits the growth and pathogenic mechanisms of several enveloped RNA viruses, and it has been observed that H_2_S plasmatic levels are elevated in COVID-19 survivors compared to fatal cases [102]. The release of H_2_S is triggered by carbon monoxide (CO) from the catabolism of heme by inducible heme oxygenase (HO-1) and heme proteins have the catalytic activity required for H_2_S signaling by protein persulfidation. Subjects with a long promoter for the HMOX1 gene, coding for HO-1, have a lower efficiency of this mechanism. SARS-CoV-2 prevents the formation of the heme of hemoglobin and other hemeproteins, so impeding both the signaling and release of H_2_S. Deficiency of the H_2_S-induced persulfidation of the K_ATP_ channels of leucocytes triggers the adhesion and release of the inflammatory cytokines, lung infiltration, and systemic endothelial damage with hyper-coagulability. These outcomes mainly justify the sex and age distribution, clinical manifestations, and co-morbidities of COVID-19 [102]. The involvement of H_2_S in COVID-19 therapy is also supported by its anti-inflammatory and antiviral functions. As is already known, H_2_S has a pivotal role in regulating the inflammatory and pro-inflammatory cytokine cascade and certain H_2_S donors are effective in the treatment of acute lung inflammation. The latest studies have elucidated the mechanism of action for the antiviral effects of hydrogen sulfide. Moreover, certain preliminary clinical results have indicated an inverse relationship between endogenous H_2_S levels and the severity of COVID-19. H_2_S donors, then, could represent a potential therapeutic opportunity for the treatment of COVID-19 [103]. The alteration of H_2_S levels in COVID-19 represent further evidence of the need to regulate the homeostasis of this gasotransmitter, considering that the alteration of H_2_S levels has an important role in several diseases [89,90,91,92,93,94,95,96,97,98,99,100,101,102,103,104].

Finally, H_2_S is especially controlled in experimental glaucoma. By scavenging reactive oxygen species and distending retinal vessels, H_2_S protects RGCs from pressure and oxidative stress both in vitro and in vivo. Consequently, H_2_S is considered to be a new neuro-protectant in glaucoma [104].

## 2. H_2_S Donors

### 2.1. Gaseous H_2_S

On the basis of the broad and active physiological effects of H_2_S, some research groups have described the protective role of H_2_S donors in various disease models. H_2_S gas has been clearly applied in these fields. The most prominent method of delivery is the direct inhalation of this gas. The delivery method has been often assessed, even though H_2_S is a toxic and flammable gas. It has been demonstrated that gaseous H_2_S generates a suspended animation-like state in mice [30] and it has been proposed that these effects are caused by the reversible and competitive inhibition of the mitochondrial enzyme cytochrome c oxidase [31]. Moreover, H_2_S gas demonstrated a reversible reduction in motor activity and body temperature with a subsequent growth in blood sulfide concentration in mice [105,106]. Consequently, inhalation of H_2_S is used as a technique to inhibit pulmonary and systemic inflammatory reactions in the course of physiological stress in hypothermic and normothermic mice [107,108]. The reversible H_2_S-induced hibernation state has been shown to protect mice from lethal hypoxia [109]. H_2_S gas can also promote glucose uptake and provide amelioration in type II diabetes [110]. H_2_S inhalation allowed a modulation of the delivery by precisely modulating pressure over time and it bypassed some of the issues with intravenous delivery as well as the oxidation and volatilization of suspended H_2_S. On the other hand, H_2_S inhalation has various inherent shortcomings because of the toxicity and flammability of the gas, including gas storage, secure administration, and targeting. From the experimental point of view, it is difficult to consider H_2_S gas as an ideal drug because of the problems in acquiring accurately regulated concentrations and for the potential toxic effect of an excess of H_2_S. 

### 2.2. Inorganic Salts 

Inorganic salts, as well as sodium hydrosulfide (NaHS), sodium sulfide (Na_2_S), and calcium sulfide (CaS), are believed to be exogenous H_2_S donors. These inorganic salts, which represent standard tools, have been long employed in biological studies to investigate the therapeutic effects of exogenous hydrogen sulfide, although several significant limits have been proved. 

Indeed, sulfide salts hydrolyze instantaneously following dissolution in water, establishing an equilibrium between hydrogensulfide ions (HS^−^), sulfide ions (S^2−^), and molecular hydrogen sulfide (H_2_S) which depend on some parameters such as pH, temperature, and pressure [111]. When this equilibrium is determined, volatilization of H_2_S appears, thus reducing the general concentration of the sulfur species in the solution. Moreover, HS air oxidation^−^, catalyzed by metals in water, lowers the concentration of H_2_S solutions [112]. These chemical limitations restrict the use of salts in clinical settings even though the administration of sulfide salts to animals, tissues, or cells has been demonstrated to have protective effects for a large number of diseases. Some researchers have highlighted the short- and long-term cardioprotective effects of H_2_S using Na_2_S as a H_2_S donor in a mouse model of myocardial ischemia injury (MI/R). The results have suggested that a 7-day treatment of Na_2_S resulted in a reduction in the dilatation of the left ventricle, a loss of cardiac hypertrophy, and an increase in cardiac function. Moreover, treatment with Na_2_S decreased the oxidative stress related to heart failure [113]. Aqueous NaHS solution delivered in aortic rings led to a 60% increase in relaxation, proving the vasorelaxant effects of H_2_S [114]; this salt reduced ischemia–reperfusion injury [115], and protected against hemorrhagic shock [116] and myocardial infarction (MI) [117]. 

Moreover, sulfide salts have protective effects for other diseases as well as inflammation [118]. Osteoarthritis (OA) is a form of arthritis characterized by degenerative and inflammatory processes and by higher levels of pro-inflammatory proteins (IL-6 and IL-8). A study reported in the literature demonstrated that the short-term treatment of human cells with NaHS is enough to down-regulate IL-6 and IL-8 expression, which in turn may cause anti-inflammatory effects of H_2_S against OA [119]. However, it has also been observed that the anti-inflammatory effects of NaHS translated into pro-inflammatory effects when the NaHS incubation time was protracted (1 h). In fact, it has been reported that sulfide salts as H_2_S donors showed both anti-inflammatory and pro-inflammatory effects. 

Some researchers have indicated that the leukocyte-regulated inflammation of the knee joint was significantly hindered after Na_2_S injection [120]. In an independent study, Chen et al. determined that the administration of NaHS into the lungs in an asthmatic rat model attenuated inflammation [71]. Through the use of a smoke inhalation-induced lung injury model, Esechie et al. proved that Na_2_S exhibited protective actions against severe lung injury [118]. These results have suggested that H_2_S (using NaHS or Na_2_S) is a potent anti-inflammatory agent. 

In contrast, the pro-inflammatory effects of H_2_S have also been highlighted. Indeed, Li et al. proved that H_2_S exhibited pro-inflammatory effects in an animal model of endotoxic shock [121]. Furthermore, plasma H_2_S concentrations, the synthesizing ability of H_2_S in tissue, and CSE expression were all proven to be enhanced in several animal models of inflammation [122,123,124]. This conclusion has indicated the potential pro-inflammatory effects of H_2_S.

In a separate study, the administration of NaHS solution by intravenous injection alleviated the degree of ALI by decreasing IL-6 and IL-8 levels while concurrently increasing IL-10 levels in the plasma and lung tissue [125]. This study also substantiated the hypothesis that the down-regulation of endogenous H_2_S levels in the cardiovascular system is present in ALI pathogenesis.

Parkinson’s disease (PD) manifests a progressive loss of dopaminergic neurons in the substantia nigra and a depletion of the neurotransmitter dopamine in the striatum [126]. Hu et al. indicated that a systemic administration of H_2_S (using NaHS) could reduce behavioral symptoms and dopaminergic neuronal degeneration in 6-hydroxydopamine (6-OHDA)-induced PD models [127]. By employing such models, they determined that endogenous H_2_S production is lowered during the development of PD, and the results have suggested that the administration of H_2_S could have therapeutic benefits for Parkinson’s disease. 

Comparatively, H_2_S has proven to be useful for Alzheimer’s disease (AD). The amount of H_2_S biosynthesis in the brain is lowered in AD patients and the levels of H_2_S in the plasma are linked to the severity of AD [44]. Giuliani et al. observed how treatment with a H_2_S donor induced neuroprotection and slowed down the progression of AD. Studies with sodium hydrosulfide and Tabiano’s spa-water were conducted in three experimental models of AD. Short-term and long-term treatments with sodium hydrosulfide and/or Tabiano’s spa-water significantly protected against impairments in learning and memory in rat models of AD induced by an injection of β-amyloid_1–40_ (Aβ) or streptozotocin in the brain, and in an AD mouse model harboring the human transgenes APPSwe, PS1M146V, and tauP301L (3xTg-AD mice). The improvement in behavioral performance was related to the size of the Aβ plaques in the hippocampus and the preservation of the morphological picture, as found in AD rats. In addition, the reduced concentration/phosphorylation levels of proteins were proven to be essential in AD pathophysiology. Specifically, the amyloid precursor protein, presenilin-1, Aβ_1-42_ and tau phosphorylated at Thr181, Ser396 and Ser202, were identified in 3xTg-AD mice treated with spa-water. The excitotoxicity-triggered oxidative and nitrosative stress was found in 3xTg-AD mice, as indicated by the decreased levels of malondialdehyde and nitrites in the cerebral cortex. The limited activity of c-jun N-terminal kinases, extracellular signal-regulated kinases, and p38 in the hippocampus presented a well-established role both in the phosphorylation of the tau protein and in inflammation and apoptosis. Consistently, the decrease in the tumor necrosis factor-a level, the up-regulation of Bcl-2, and the down-regulation of BAX and the downstream executioner caspase-3 in the hippocampus of 3xTg-AD mice after treatment with Tabiano’s spa-water implied that it is also able to modulate inflammation and apoptosis. These results indicate that appropriate treatments with H_2_S donors and Tabiano’s spa-waters and other spa-waters rich in H_2_S content might represent an innovative approach to slow down AD progression in humans by targeting multiple pathophysiological mechanisms [128]. 

The low pharmacokinetic profile of sulfide salts limits the use of these salts as potential therapeutics and has led to the research into innovative organic H_2_S donors, including naturally occurring donors and synthetic compounds.

### 2.3. Natural H_2_S Donors

**Figure 4:**
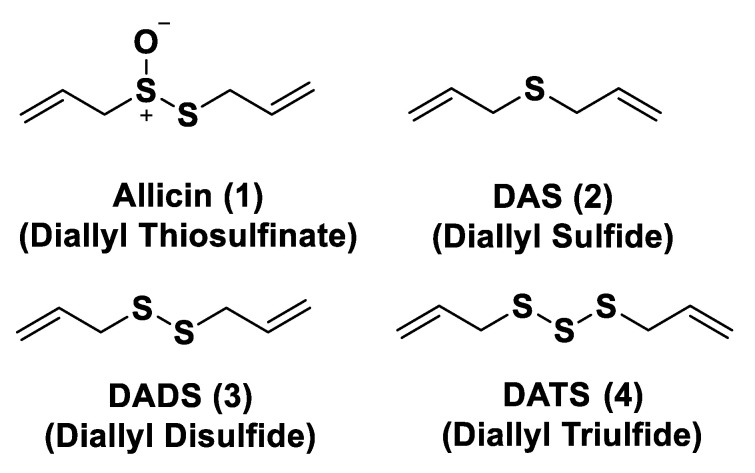
Chemical structures of some garlic-derived sulfur compounds.

As is already known, garlic and onions contain substances that have benefits for the prevention or treatment of several diseases such as cardiovascular disease, hypertension, thrombosis, and diabetes [129]. H_2_S-releasing compounds from garlic extracts seem to be essential elements; in particular, these compounds are byproducts that originate from metabolism of thiosulfinates (R–SO_2_–SR) [130]. The integral garlic contains γ-glutamil-S-alk(en)yl-l-cysteines that undergo hydrolysis and oxidation reactions which lead to alliin; the latter is converted into allicin through a reaction catalyzed by alliinase. Allicin (**1**) represents the main sulfur compound and the most common thiosulfinate; the instant decomposition of this compound in aqueous media leads to some oil-soluble compounds such as diallyl sulfide (DAS, **2**), diallyl disulfide (DADS, **3**), and diallyl trisulfide (DATS, **4**) (Figure 4) [131]. γ-glutamil-S-alk(en)yl-l-cysteines can also lead to water soluble organo-sulfur compounds including S-allyl cysteine (SAC) and S-allyl mercaptocysteine (SAMC). 

Research on DAS, DADS, and DATS has shown that human red blood cells transform these compounds into H_2_S in the presence of free thiols and that treatment of aortic rings with these compounds leads to vasorelaxation, similar to experiments using sulfide salts [49]. Several polysulfide species have also been extracted from a variety of other plants; however, different from garlic-derived donors, they have not currently been evaluated [132]. Many studies reported in literature regarding the garlic-derived H_2_S donors have focused on the mechanisms through which these donors allow the release of H_2_S in the presence of thiols. Benavides et al. reported that DADS and DATS can quickly release H_2_S by means of a thiol-dependent mechanism [49]. In particular, in the presence of GSH, DADS leads to S-allyl glutathione (GSA) and the intermediate allyl perthiol (ASSH), which reacts with GSH, thus producing H_2_S and S-allyl glutathione disulfide (GSSA). Moreover, this compound can also undergo α-carbon nucleophilic substitution, swiftly generating H_2_S. This mechanism has been proven to be valid, except for DADS. Concerning this aspect, it has been shown that DADS releases H_2_S very slowly thanks to an α-carbon nucleophilic substitution [133]. The previous misunderstanding, (DADS as a fast H_2_S donor) was due to DATS contamination in commercial samples of DADS; indeed, among these compounds, DATS is responsible for the rapid H_2_S release. DATS can give two possible thiol−disulfide exchange reaction pathways: primarily, the nucleophilic attack of GSH on allylic sulfur of DATS that generates GSSA and ASSH, with the latter possibly reduced by GSH and then releasing H_2_S; further, the nucleophilic attack of GSH on the central sulfur atom of DATS can lead to the production of ASH and GSSSA. The latter rapidly produces hydrogen sulfide. 

Another class of natural H_2_S donors is composed by some isothiocyanates (Figure 4); these compounds represent natural donors that have attracted attention due to their biological and pharmacological effects in the prevention of important human diseases, as well as cancer, neurodegenerative processes, and cardiovascular diseases [134]. The isothiocyanate moiety has been thoroughly investigated due to its use in synthetic molecules for structure activity relationship studies. In a recent study, the hydrogen sulfide-releasing properties of some synthetic aryl isothiocyanate derivatives were reported, indicating that the isothiocyanate function can be considered as a suitable slow hydrogen sulfide-releasing moiety, along with the pharmacological potential typical of this gasotransmitter. Many isothiocyanate derivatives (as a result of the myrosinase-mediated transformation of glucosinolates) are well-known secondary metabolites of plants belonging to the family Brassicaceae, a large botanical family comprising of many edible species. The phytotherapeutic and nutraceutic usefulness of Brassicaceae in the prevention of important human diseases, such as cancer, neurodegenerative processes, and cardiovascular diseases has been widely discussed in the scientific literature. Although these effects have been associated with isothiocyanates, by and large, the exact mechanism of action is still unknown.

**Figure 5:**
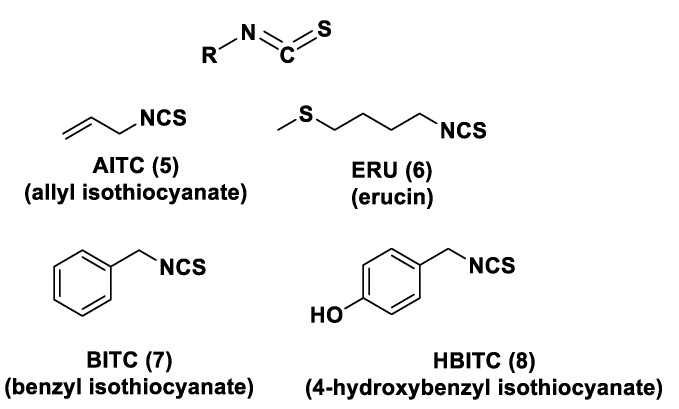
General structure of isothiocyanates and the chemical structures of natural isothiocyanates tested in [135].

In a recent experimental study, the hydrogen sulfide-releasing capacity of some important natural isothiocyanates has been investigated (AITC (**5**), ERU (**6**), BITC (**7**), HBITC (**8**)—Figure 5) by using in vitro amperometric detection. Some of the tested natural isothiocyanates exhibited a significant release of hydrogen sulfide, thus implying that hydrogen sulfide may be, at least in some measure, a relevant player accounting for several biological effects of Brassicaceae [135].

In addition, it has been proven that some selected aryl isothiocyanates are able to release H_2_S in a biological environment and produce a vasorelaxant effect in rat aortic rings, strongly antagonized by a specific Kv7-blocker, and in the coronary vascular bed, causing an increase in the basal coronary flow [136]. Furthermore, the H_2_S-releasing properties of the secondary metabolites of Brassicaceae, isothiocyanates, are also derived from the quick reaction with thiols, namely cysteine. The Cys-ITC adduct formed undergoes an intramolecular cyclization followed by a release of an organic amine R−NH_2_ and raphanusamic acid (RA), on the one hand, and H_2_S and 2-carbylamino-4,5-dihydrothiazole-4-carboxylic acid, on the other hand [137].

Although naturally occurring H_2_S donors represent an attractive tool for in vivo studies, the tendency of these compounds to also bring about some byproducts, which are not related to H_2_S production, has prompted researchers to develop novel synthetic H_2_S donors with more favorable pharmacokinetic and safety profiles. 

Indeed, synthetic donors, which provide tunable H_2_S release rates via structural modifications with discrete byproducts, allow for a more in-depth analysis of the physiological roles of H_2_S and, optimistically, clinically relevant H_2_S-releasing prodrugs.

In this review, H_2_S donors are presented according to their main role in several contexts: cardiovascular diseases, cancer, nervous system diseases, gastrointestinal diseases, dermatological diseases, viral infections, and other diseases.

## 3. H_2_S Donors and Diseases

### 3.1. H_2_S Donors and Cardiovascular Diseases

As already reported, the current scientific literature has proven the protective actions of H_2_S in cardiovascular diseases, for instance hypertension, atherosclerosis, cardiac hypertrophy, heart failure, and myocardial ischemia/reperfusion (I/R) injury. The involvement of H_2_S in the cardiovascular system is due to its role as a blood pressure and heart rate regulator, an activator of angiogenesis, and a basal vasorelaxant agent. Furthermore, it has been proven that antioxidation, the inhibition of cell apoptosis, pro-angiogenesis, the anti-inflammatory effect, and the regulation of ion channels represent the mechanisms of action that lead to cardioprotective activity [95].

Based on the involvement of H_2_S in the cardiovascular system, scientific research has shown a strong interest in the development of H_2_S donors, which could represent biological instruments and encouraging cardioprotective agents.

#### 3.1.1. Lawesson’s Reagent and Derivatives

**Figure 6:**
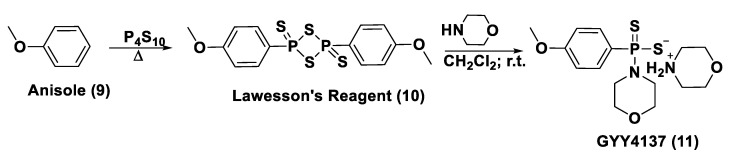
Chemical synthesis of Lawesson’s Reagent (**10**) and GYY4137 (**11**).

Lawesson’s reagent (2,4-Bis(4-methoxyphenyl)-1,3,2,4-dithiadiphosphetane-2,4-disulfide—LR, **10**) is an extensively applied sulfurization reagent in organic synthesis [138] and a H_2_S donor. LB is simply synthesized through a reaction between anisole (**9**) and phosphorus pentasulfide (P_4_S_10_) (Figure 6) [139,140]. 

LR is a known, commercially available compound used for the thionation of alcohols, ketones, esters, and amides to obtain the sulfur analogs [138]. In aqueous media, this compound releases H_2_S through a hydrolysis release mechanism and the release time is greater than that of the sulfide salts.

It has been seen that the oral administration of Lawesson’s reagent, preceding alendronate-induced gastric damage, restricted successive gastric impairment [141]. An increase in GSH levels in rats handled with LR that can be ascribed to the reduction in oxidative stress and inhibition of neutrophil infiltration has also been observed. In a different in vivo study, exogenous H_2_S delivery from LR increased leukocyte adhesion and neutrophil migration to the area where sepsis was stimulated in mice, increasing the global survival probability of the affected mice [142]. Moreover, it was observed that the mortality rate of the mice during the experiment was improved, with a reduction in endogenous H_2_S production using a CSE inhibitor. Nevertheless, LR releases H_2_S through spontaneous and uncontrollable hydrolysis in aqueous solution. This kind of H_2_S release and the poor solubility of Lawesson’s reagent in aqueous solutions restricts its applications.

GYY4137 (Morpholin-4-ium-4-methoxyphenyl-(morpholino)-phosphinodithioate; **11**), is a water-soluble Lawesson’s reagent derivative and one of the first slow-releasing H_2_S donors developed [143,144,145]. This reagent is the compound that is most used to examine the role of H_2_S in the biological districts. It can be synthesized by reacting Lawesson’s reagent [139,140] with morpholine in methylene chloride at room temperature (Figure 6) [143].

GYY4137 exhibits anti-inflammatory, antioxidant, and anticancer properties [146]. In addition, this reagent triggers the activation of the vascular smooth muscle K_ATP_ channels and relaxes rat aortic rings and the renal blood vessels that lead to anti-hypertensive activity [143]. Furthermore, GYY4137 interfered with platelet activation and adhesion molecule-mediated aggregation reducing the formation of the microvascular thrombus and stabilizing atherosclerosis plaque [147]. It seems to protect against diabetic myocardial I/R injury (activating the PHLPP-1/Akt/Nrf2 pathway) [148] and against cerebral I/R injury (via the p38 MAPK mediated anti-apoptotic signaling pathways) in rats [149]. It has been proven that GYY4137 slowly releases H_2_S upon hydrolysis. To explain the two-step hydrolytic decomposition pathway of the slow-release H_2_S donor GYY4137, a combination of NMR spectroscopy, mass spectrometry, and chemical synthesis has been used and the key decomposition product has been also obtained by an independent synthetic strategy. The (dichloromethane-free) sodium salt of the phosphonamidodithioate GYY4137 has also been made as a pharmaceutically more appropriate salt. It has been shown that the decomposition product does not produce H_2_S and does not have cytoprotective or anti-inflammatory effects in oxidatively stressed human Jurkat T-cells and LPS-treated murine RAW264.7 macrophages, in contrast with GYY4137 and its sodium salt. The decomposition product signifies a helpful control compound for establishing the biological and pharmacological effects of H_2_S generated from GYY4137. Whilst GYY4137 certainly represents a useful biological compound, it presents some disadvantages that mean the attribution of biological effects to GYY4137-derived H_2_S is undefined because of the probable concurrent metabolization of dichloromethane to CO, a gasotransmitter with biological effects similar to H_2_S [150,151].

**Figure 7:**
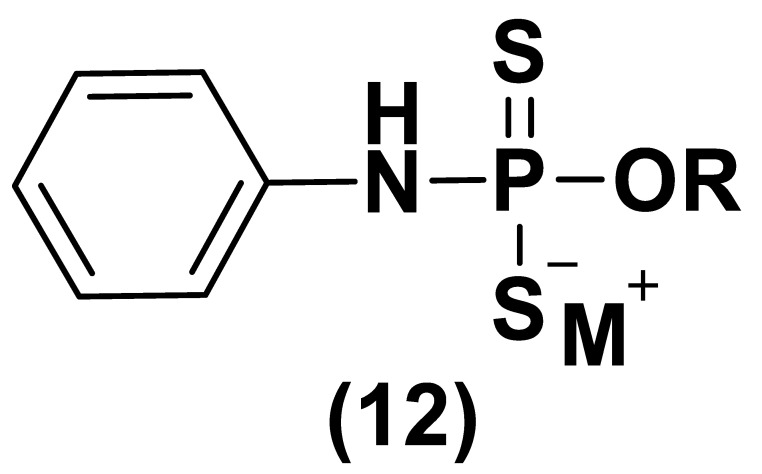
General chemical structures of O-aryl and O-alkyl GYY4137 derivatives.

With the aim to overcome the described undesired events, structural modifications of GYY4137 have been designed and the subsequent analogs have been synthesized and examined. O-aryl- and alkyl-substituted phosphorodithioates (**12**) were designed as H_2_S donors by switching the P-C bond in GYY4137 with O-substitution (Figure 7) [152]. These H_2_S donors did not significantly release hydrogen sulfide. In fact, O-arylated donors demonstrated a slow production of H_2_S, while O-alkylated donors showed very low H_2_S generation.

**Figure 8:**
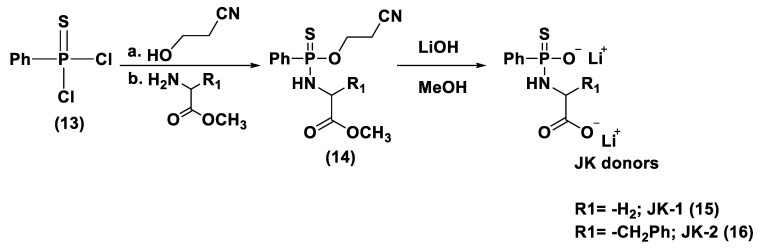
Synthesis of JK donors.

Bearing in mind the GYY4137 structure, JK donors (**15**, **16**), a novel kind of phosphonamidothioate, were obtained [152]. The synthesis of these compounds started from the treatment of phenylphosphonothioic dichloride (**13**) with 3-hydroxypropionitrile and, subsequently, with a selected C-protected amino acid (as well as glycine, phenylalanine, valine, alanine, and proline) in the presence of triethylamine. The obtained intermediate (**14**), after a LiOH-mediated hydrolysis in MeOH, provided the desired compounds (**15**, **16**) (Figure 8).

JK donors are the first class of pH controllable H_2_S donors. These compounds give higher and faster H_2_S releasing rates in aqueous media and under acidic conditions, while they have slower and lower release rates at a neutral and mildly basic pH. These donors are characterized by a novel mechanism of release, characterized by the protonation of phosphonamidothioates that give the corresponding phosphorothiols and that, after the cyclization via nucleophilic addition of the carboxylic acid group, lead to the breaking of the P-S bond and to H_2_S release. JK-1 and JK-2, the two main donors of the series (Figure 8), had protective effects on cellular and murine models of myocardial I/R injury and significantly reduced infarct size and the circulating troponin-I level [153].

Ammonium tetrathiomolybdate ((NH_4_)_2_MoS_4_, ATTM) is a copper chelator and represents another pH dependent H_2_S donor [154]. It is able to release H_2_S even under strong acidic conditions (5% H_2_SO_4_).

ATTM is a water-soluble and slow releasing H_2_S donor; studying the gaseous release in different pH buffers (from 5 to 8), it has been shown that the level of H_2_S at pH 5 is considerably higher than the levels at other pH values [155]. Furthermore, it has been observed that H_2_S concentrations were stable for long time in all solutions, demonstrating that ATTM is a stable and slow releasing H_2_S donor.

ATTM had protective effects on the heart and brain in rat models of myocardial and cerebral I/R injury and reduces I/R injury [156]. In fact, certain studies are currently evaluating the potential dual role of ATTM as a cardioprotective and anticancer agent in patients with cancer treated with doxorubicin or Adriamycin [157,158,159]. Moreover, ATTM has recently progressed in clinical trials for the treatment of breast cancer because of its copper depletion effects [160].

#### 3.1.2. DTTs

**Figure 9:**
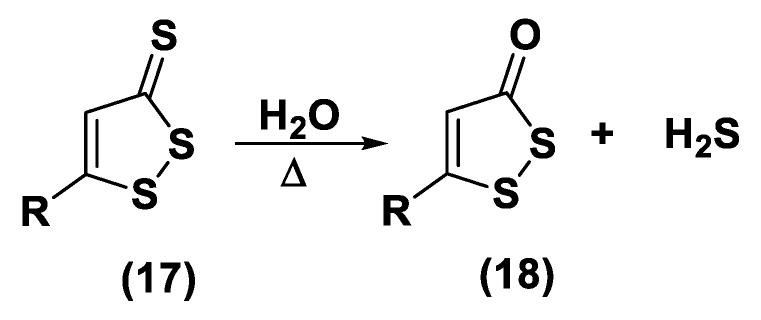
H_2_S release from DTTs.

DTTs (1,2-Dithiole-3-thiones, **17**) are another class of hydrolysis-triggered H_2_S donors. Even if their H_2_S-releasing mechanism is not totally delineated, it is commonly agreed that the production of H_2_S from DTTs occurs via hydrolysis (Figure 9).

It has been proven that DTT derivatives progressively release H_2_S, converting it into the corresponding 1,2-dithiole-3-one (**18**) with heating to 120 °C and in aqueous solution [161].

**Figure 10:**
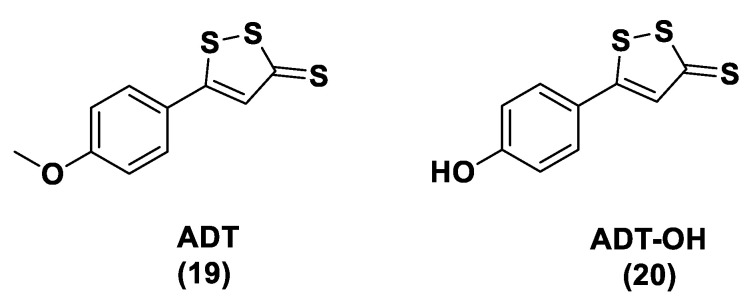
Chemical structures of ADT and ADT-OH.

ADT (Anethole dithiolethione, **19**) and ADT-OH (5-(p-hydroxyphenyl)-3H-1,2-dithiole-3-thione, **20**), a O-demethylated ADT derivative, are the most employed DTTs as H_2_S donors (Figure 10).

In terms of the mechanism of H_2_S release by this class of compounds, it must be specified that to date not all the steps, metabolic and/or chemicals, have been elucidated. In particular, important information comes from the observation that S-diclofenac releases H_2_S in a time-dependent manner in both rat plasma and liver homogenates. This implies a metabolic step as the initiation of the release mechanism. A similar result is also obtained by the incubation of ADT-OH. To date, the nature of this activation step, which does not involve enzymes belonging to the esterase class, remains unidentified [162].

**Figure 11:**
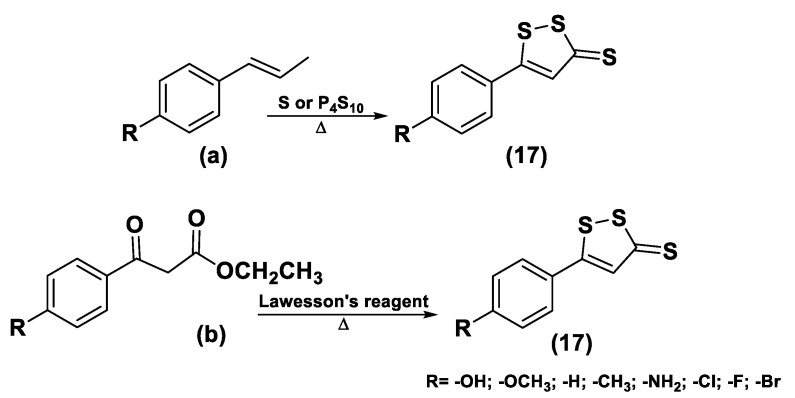
Examples of synthetic strategies used to obtain DTTs.

The synthesis of DTTs is easy and provides the reaction of anethole with elemental sulfur; these compounds can also be easily added to other molecules to obtain drug–DTT conjugates. Nevertheless, different synthetic methods have been proposed for their preparation [163]. The most used synthetic strategy is based on the dehydrogenation and sulfurization of an allylic methyl group (**a**) which involves treatment with elemental sulfur or phosphorus pentasulfide (Figure 10). On the other hand, β-ketoesters (**b**) have been reacted with Lawesson’s reagent to provide the requested DTTs (**17**) [142,164,165,166] (Figure 11).

Several studies have reported the biological effects and the activity of ADT and ADT-OH correlated with the H_2_S-releasing properties. In particular, ADT represents an FDA approved drug; this compound is able to promote bile secretion, restoring salivation and reducing dry mouth in patients that present with chemotherapy-induced xerostomia [167]. ADT-OH indeed has been proven to decrease cell viability through the inhibition of histone deacetylase [168,169] and NF-kB activation [170].

**Figure 12:**
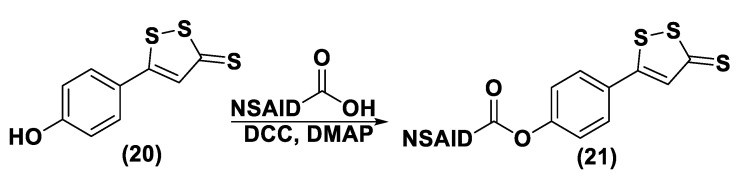
General synthetic strategy used to obtain HS–NSAIDs.

H_2_S donor hybrids (**21**) have been achieved through the copulation reaction between the hydroxylic group of ADT-OH (**20**) with commercially available drugs and the obtaining compounds have been examined for their H_2_S-releasing properties and therapeutic activities [12]. The reaction between ADT-OH and active compounds, in the presence of *N,N*’-dicyclohexylcarbodiimide (DCC) and 4-dimethylaminopyridine (DMAP), generally leads to the production of the considered H_2_S donor hybrids (Figure 12) [162].

**Figure 13:**
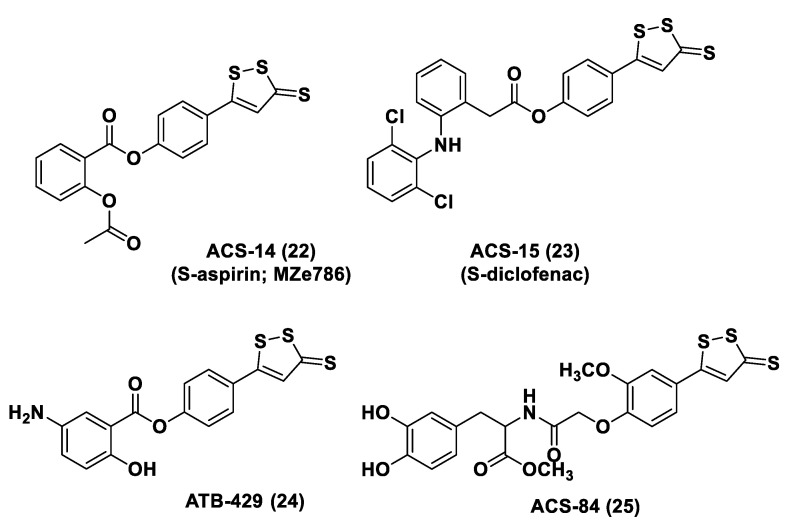
Chemical structures of representative H_2_S-NSAIDs.

Non-steroidal anti-inflammatory drugs (NSAIDs), as well as ibuprofen and aspirin, have been broadly applied for inflammation treatment [171]. Some studies have revealed that the unpleasant effects induced by NSAIDs (for example, stomach bleeding and gastrointestinal ulceration) can be substantially diminished by combining DTT molecules with the parent NSAIDs [12]. For example, a H_2_S-aspirin hybrid has been synthesized (ACS-14, S-aspirin, MZe786 (**22**); Figure 13) and the negative gastric effects caused by ACS-14 and aspirin in rats have been compared [172]. It has been shown that after oral administration of aspirin for 7 days, an extensive hemorrhagic lesion in the stomach of rats occurred. By contrast, rats given an equivalent dose of ACS-14 did not present with gastric damage. Then the H_2_S levels in plasma before and after ACS-14 were compared, and the obtained results revealed that drug administration led to a three-fold increase in the H_2_S concentration in the plasma. These results indicate that the advantages of ACS-14 in gastric protection are probably correlated with the increase in H_2_S formation. ACS-14 has also shown very important protective activities in pathological cardiovascular alterations in rats induced by a GSH-synthase inhibitor (BSO, buthionine sulfoximine) by improving systolic blood pressure and reducing heart rates. Furthermore, BSO-induced hypertensive effects were reduced by oral administration of ACS-14, unlike aspirin which did not have this effect. Additionally, administration of ACS-14 decreased myocardial I/R injury in BSO rats [173,174,175].

Hypertensive disorder in pregnancy is a main cause of maternal and perinatal mortality. Soluble Flt-1 (sFlt-1) is a protein whose values are elevated in preeclampsia and may continue to be high during postpartum in women with a preeclampsia diagnosis. Heme oxygenase-1 (Hmox1/HO-1) is another protein that has protective effects versus oxidative stimuli and is altered in the placenta of pregnant women with preeclampsia. Ahmed et al. assumed that soluble Flt-1 impeded cardiac mitochondrial activity in HO-1-deficient mice. It was demonstrated that the deprival of HO-1 perturbed cardiac mitochondrial respiration and decreased mitochondrial biogenesis. The overexpression of sFlt-1 caused the inhibition of cardiac mitochondrial activity in Hmox1^+/^^−^ mice. This research revealed that MZe786 restored mitochondrial activity through the stimulation of cardiac mitochondrial biogenesis and antioxidant protection in Hmox1^+/−^ mice and in Hmox1^+/−^ mice subjected to a high sFlt-1 environment [176,177]. Moreover, Ahmed et al. determined the therapeutic value of MZe786 in HO-1 haploid-deficient (Hmox1^+/−^) pregnant mice in a high sFlt-1 environment. It was demonstrated that the animal model used presented critical preeclampsia symptoms and a decrease in antioxidant genes. MZe786 reduced preeclampsia-stimulating antioxidant genes and then reduced hypertension and renal damage. MZe786 was also better for fetal outcomes in comparison with aspirin alone and was shown to be a good therapeutic agent in preventing preeclampsia [178].

Moreover, H_2_S-releasing diclofenac (ACS-15, S-diclofenac (**23**); Figure 13) has been investigated. In some studies ACS-15 demonstrated an enhanced anti-inflammatory effect in comparison with diclofenac. ACS-15 considerably decreased the lipopolysaccharide-induced infiltration of neutrophils into the liver and the lungs without increasing leukocyte adherence, as instead diclofenac [66,171].

ATB-429 (mesalamine derivative (**24**); Figure 13) represents a hybrid drug from mesalamine that has been used in animal models of Crohn’s disease and ulcerative colitis. In preclinical studies concerning the visceral pain associated with inflammatory bowel disease, this compound was more successful than mesalamine [86,179].

Beside NSAIDs, other DTT-H_2_S donor hybrids have been developed. As is already known, L-DOPA represents a drug used in the clinical treatment for Parkinson’s Disease and dopamine-responsive dystonia. Some side effects of long-term L-DOPA treatment as well as dyskinesia seem to be correlated to the oxidative stress that leads to the loss of neurons [180,181]. Because H_2_S diminishes oxidative stress, L-DOPA coupled with a H_2_S-releasing moiety could lead to improved bioactivity. In fact, Lee et al. synthesized DOPA–DTT conjugates and studied their antioxidant effects [182]. The hybrid compounds (such as ACS-84 (**25**); Figure 13) released H_2_S in the mitochondria. Additionally, this compound has been shown to inhibit MAO B, the dopamine metabolizing enzyme; therefore, it could be useful to reestablish the Parkinson’s Disease-depleted dopamine levels in the brain.

**Figure 14:**
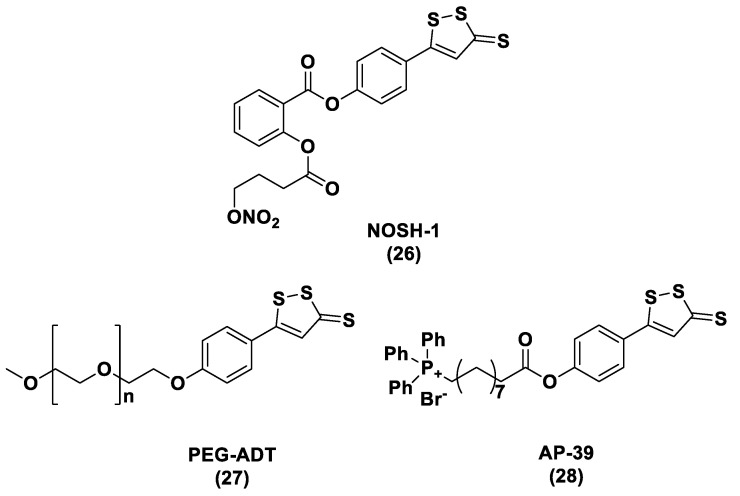
Chemical structures of representative NO-H_2_S-releasing hybrids, PEG-ADT, and AP-39.

NO-NSAIDs represent another class of hybrid drugs well studied where NO is released [183] (Figure 14). NO and H_2_S share several comparable actions, and new NO–H_2_S-releasing hybrids (for example NOSH-1(**26**)) have been designed and studied (Figure 13) [174,184,185]. These compounds are able to induce higher NO and H_2_S concentrations in vivo and have shown important activities related to the inhibition of the growth in cancer cell lines and anti-inflammatory activities in animal models.

Recently, DTT-based polymeric H_2_S donors have been reported [186]. DTT has been conjugated to poly(ethylene glycol) polymer, obtaining PEG-ADT (**27**; Figure 14) that represents a H_2_S donor that demonstrates a lower and slower H_2_S release when compared to DTT alone, probably as a result of the steric hindrance of PEG. PEG-ADT has also been shown to have fewer toxic effects as compared to the parent donor compound; this important characteristic is probably due to the co-localization of the PEG-ADT conjugate within the endo-lysosomes without escaping to the cytoplasm. It is important to consider that even if DTTs have been asserted as H_2_S donors, it is not clear if their effects are related to H_2_S; in fact, DTTs can considerably improve GSH levels in vivo by inducing the enzymes involved in the preservation of the balance of GSH [187]. It is probable that the higher GSH level contributes much more than H_2_S to those pharmaceutical effects.

AP-39 (**28**) is another mitochondria-targeted H_2_S donor hybrid, obtained from the reaction of triphenylphosphonium (TPP) with ADT-OH [188]; it has been investigated for its H_2_S-releasing properties and pharmacological effects (Figure 14) [189]. After being rapidly up-taken and accumulated in a mitochondrial membrane-potential (ΔΨ) dependent-manner, the AP-39-induced H_2_S release in the mitochondria was confirmed by several fluorescent probes. It was also found that AP-39 significantly inhibits the oxidative stress-induced toxicity by silencing the mitochondrial cell death pathways and consequently contributing to the maintenance of cell homeostasis. Furthermore, in vivo studies showed that AP-39 is useful in acute cardiac arrest, renal, and myocardial I/R injury, through the inhibition of mitochondrial permeability [190,191,192,193]. Following the development of AP-39, several similar TPP-conjugated H_2_S releasing compounds were developed by the same research group, namely AP-123 and RT01 [194]. It is important to mention that despite the numerous beneficial effects that AP-39 was able to demonstrate, in certain pathologies such as cardiac ischemia/reperfusion injuries where the therapeutic window for corresponding pharmaceutical intervention is very narrow, the very slow rate of H_2_S release from AP-39 might not be the main protective reaction mechanism. In addition, the ten-carbon long aliphatic linker incorporated in the structure of AP-39 makes this molecule hydrophobic enough to hypothesize that its incorporation and long-lasting presence in the lipophilic membranes results in the very limited effect on specific molecular targets.

DTTs are also known to take part in other biological reactions, for example, electrophilic reactions [195] and the formation of reactive radicals [196].

#### 3.1.3. Thiol-Activated H_2_S Donors

##### N-Mercapto-Based Donors

**Figure 15:**
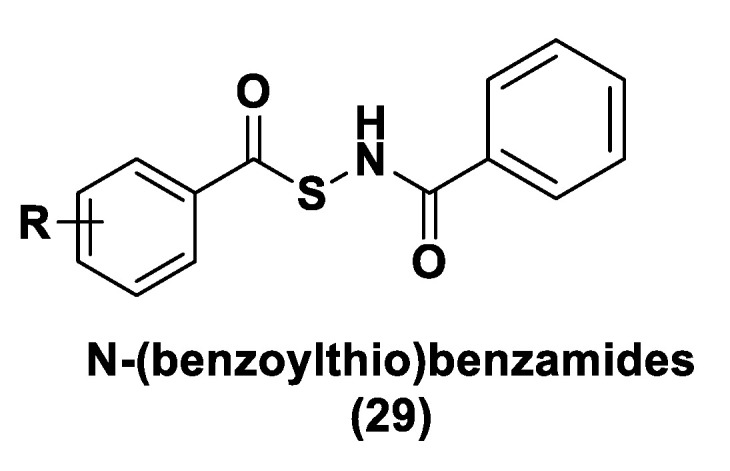
General chemical structure of N-(benzoylthio)benzamides.

In 2011, on the basis of the structure of N-mercapto (N-SH) compounds, the first thiol-activated H_2_S donors were reported [197]. As is well known, the N-SH bond is unstable and can be simply broken, providing a hydrosulfide anion. This feature led to the design of controllable H_2_S donors based on the N-SH scaffold. The synthesis of these compounds was conducted using acyl groups to protect the thiol residue of N-SH. The obtained N-(benzoylthio)-benzamides (**29**) is stable in aqueous buffers and is able to generate H_2_S in the presence of thiols (cysteine or glutathione) (Figure 15).

Structural activity relationship studies carried out in PBS buffers of a series of such donors revealed that structural modifications can modify the release of H_2_S from these compounds. In particular, the presence of electron withdrawing groups promoted faster H_2_S generation, but electron-donating groups slowed down the release of H_2_S. Thiol-activated H_2_S donors have been also assessed in plasma and similar results in H_2_S release have been obtained. 

**Figure 16:**
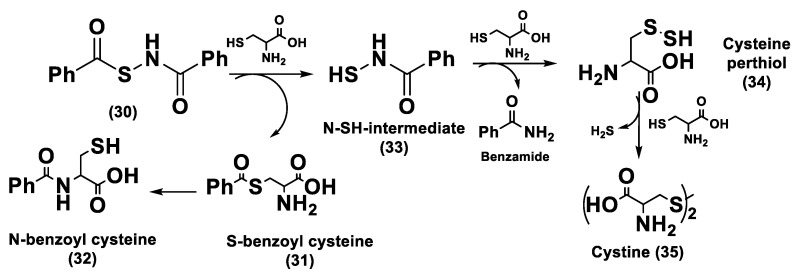
H_2_S release from N-mercapto-(N-SH) compounds.

The mechanism of H_2_S release from this N-mercapto-(N-SH) has been reported (Figure 16). Firstly, a thioester (**30**) exchange between the donor and cysteine occurs. The resultant S-benzoyl cysteine (**31**) forms a more stable N-benzoyl cysteine (**32**) due to a fast S to N acyl transfer. The N-SH intermediate (**33**) instead reacts with another molecule of cysteine to produce benzamide and cysteine perthiol (**34**); the latter reacts with cysteine to give cysteine disulfide (cystine, **35**) and releasing H_2_S.

##### S-Aroylthiooximes

**Figure 17:**
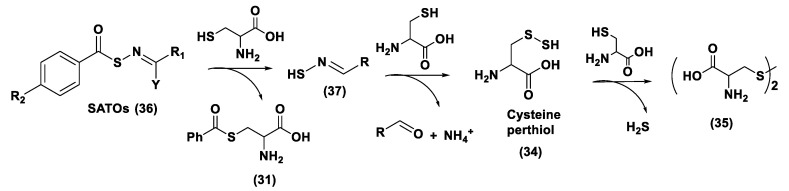
H_2_S release from SATOs.

N-SH-based H_2_S donors bearing an S-aroylthiooxime (SATO) moiety represent another class of H_2_S donors (**36**, Figure 17) [198]; this class of compounds has been obtained through a reaction between substituted S-aroylthiohydroxylamines (SATHAs) and ketones or aldehydes. Under a physiological pH, these compounds have proven to be moderately stable in aqueous solutions and their hydrolysis rate could be monitored by modulating the electronic and steric factors. H_2_S half-life, obtained from thiols, is between 8 and 82 min and can be modified by substituent variations on the SATHA ring. The H_2_S-releasing mechanism for these new molecules is comparable to that of N-(benzoylthio) benzamides; in fact, it starts with a reversible thiol exchange between the SATOs (**36**) and cysteine to form the arylidenethiooxime (**37**) and S-benzoyl cysteine. The subsequent reaction of another molecule of cysteine with the arylidenethiooxime (**37**) produces cysteine perthiol (**34**), aldehyde, and ammonia. Lastly, cysteine perthiol (**34**) reacts with cysteine to give H_2_S and cystine (**35**).

##### Perthiol-Based Donors

Since cysteine perthiol represents an important compound for H_2_S generation from N-mercapto-based donors, perthiols have been considered for the design of novel H_2_S donors. Indeed, cysteine perthiol is implicated in H_2_S biosynthesis that is catalyzed by CSE and it can be considered a “natural” H_2_S donor [12,41].

**Figure 18:**
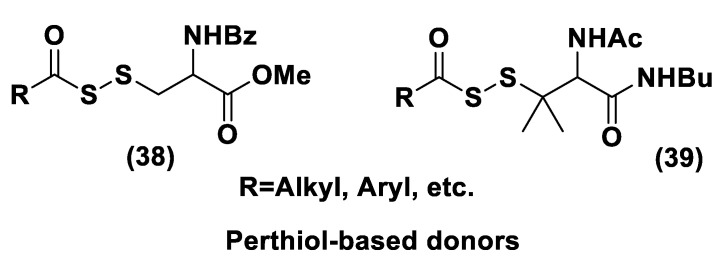
General chemical structures of perthiol-based H_2_S donors.

Considering this feature, a class of perthiol-based donors have been synthesized as cysteine (**38**) and penicillamine (**39**) derivatives (Figure 18) [199]. To protect the thiol residue, acyl groups were used. The evaluation of the H_2_S-releasing profile of these donors was carried out in the presence of thiols and in aqueous buffers and showed that cysteine-based donors release very small amounts of hydrogen sulfide (probably caused by an undesirable disulfide cleavage of the donors by thiols that leads to no H_2_S production), while penicillamine-based donors have a high efficiency in H_2_S generation. Probably the presence of two adjacent methyl groups precluded the cleavage of the disulfide bonds by thiols and then the diacylation and following H_2_S production dominated in the reaction leading to a better H_2_S production.

Studies concerning the mechanism of H_2_S release from these donors showed similarities to that of N-mercapto-based donors.

H_2_S release from penicillamine-derived perthiol donors in H9c2 cells and in in vivo studies has been also evaluated. The cardioprotective effects of a number of compounds have been also observed in a myocardial ischemia/reperfusion (MI/R) injury murine model. The results revealed that these donors had therapeutic benefits reducing the infarct size in MI/R mice.

##### Dithioperoxy-Anhydrides

**Figure 19:**
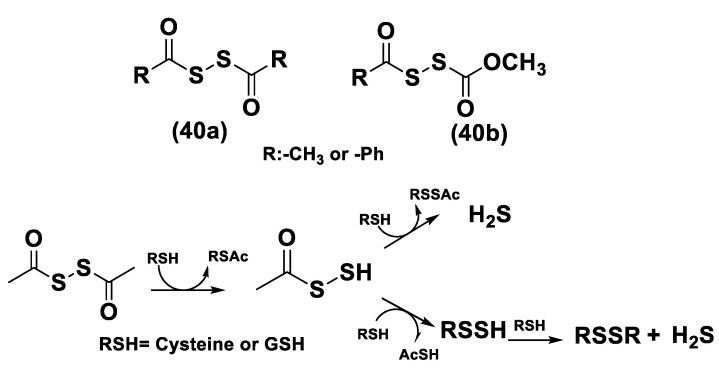
Chemical structures and the H_2_S release mechanism of dithioperoxy-anhydrides.

Dithioperoxy-anhydrides (**40a**, **40b**) represent another kind of thiol-activated H_2_S donor [200]. Considering the disulfide linkage, these donors have some chemical similarities with perthiol-based donors. Acylpersulfides are very important intermediates for H_2_S release and two probable pathways have been considered: (a) a direct reaction between acylpersulfides and thiols to form H_2_S and RSSAc; (b) the production of a new perthiol leading to H_2_S and disulfide generation (Figure 19).

H_2_S release has been proven in both buffers and HeLa cell lysates. Moreover, the CH_3_C(O)SSC(O)CH_3_ compound is effective in the concentration-dependent vaso-relaxation of a pre-contracted rat aortic ring through its H_2_S release.

##### Thioamide- and Aryl Isothiocyanate-Based Donors

**Figure 20:**
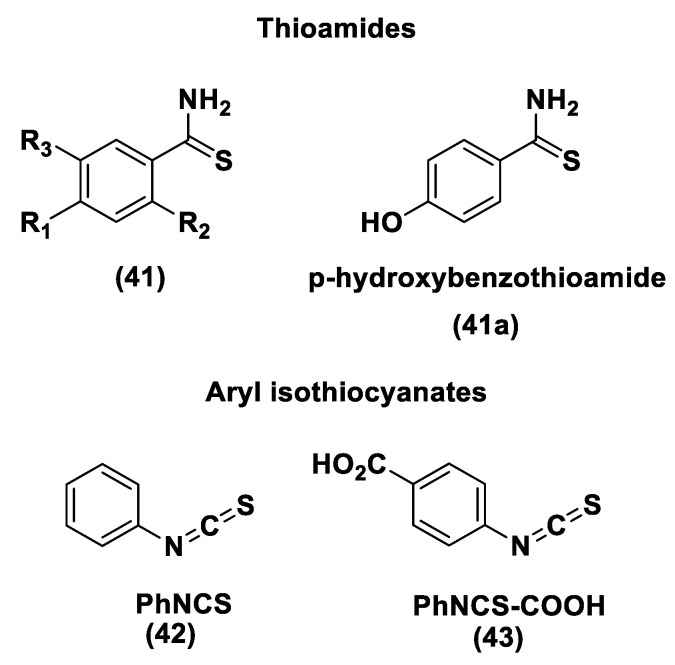
Chemical structures of thioamide- and aryl isothiocyanate-based donors.

Arylthioamide derivatives (**41**) represent a class of thiol-activated H_2_S donors, even if these compounds are weak and slow-releasing donors (Figure 20) [146].

To evaluate the vasoconstriction effects, arylthioamides have been tested; it was observed that the pretreatment of aortic rings with compound **41a** (p-hydroxybenzothioamide), considerably inhibited the vasoconstriction generated by noradrenaline. Oral administration of p-hydroxybenzothioamide in rats led to a decrease in blood pressure, supporting the protective actions of this donors in cardiovascular systems. Endogenous thiols were sufficient to promote H_2_S release from the donors because neither exogenous cysteine nor glutathione were added.

Aryl isothiocyanate derivatives represent another class of slow-releasing H_2_S donors (Figure 20) [136]. Thiol activation for these derivatives proved to be required. PhNCS (**42**) and PhNCS-COOH (**43**) exhibited vaso-relaxing effects on conductance and coronary arteries but the biological mechanism is unclear. It has been proposed that aryl isothiocyanates are able to react with biomolecules (for example cysteine) to produce thioamide compounds and then H_2_S.

##### *gem*-Dithiol-Based H_2_S Donors

**Figure 21:**
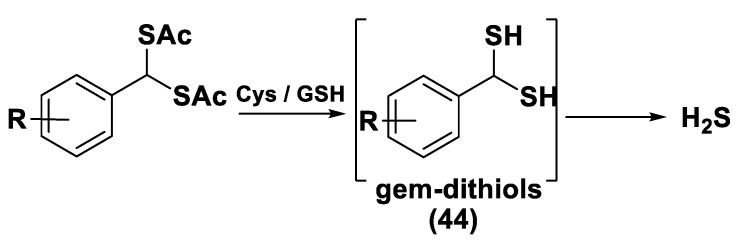
H_2_S release from acylated *gem*-dithiols.

*gem*-Dithiols represent unstable compounds in aqueous solutions and H_2_S is the decomposition product [201,202]. They belong to thiol-activated H_2_S donors (**44**, Figure 21) [145,203]. These compounds have been modified by acylation and a library of donors have been obtained. H_2_S release from these donors has been determined in the presence of Cys or GSH. Donor-induced H_2_S release has been similarly examined in HeLa cells (in the presence of endogenous thiols).

#### 3.1.4. Enzyme-Triggered Donors

**Figure 22:**
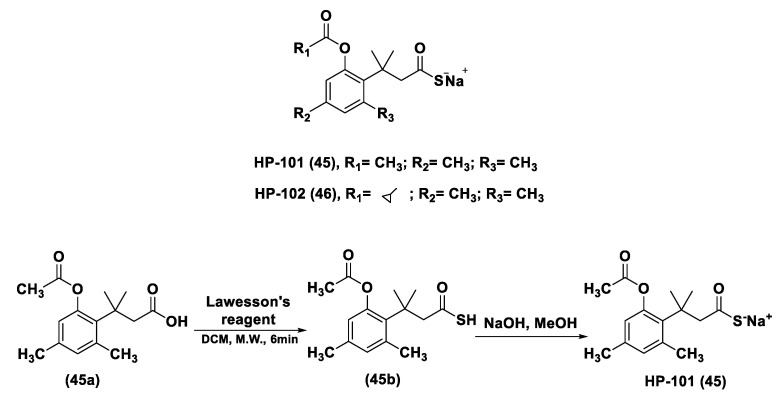
TML chemical structures and the synthetic route for HP-101 preparation.

In 2016 Zheng et al. developed a series of esterase-sensitive prodrugs, based on a lactonization reaction, otherwise known as a ‘‘trimethyl lock” (TML) [204,205]. HP-101 (**45**) was the first obtained derivative (Figure 22) and it was synthesized under microwave conditions starting from compound **45a** by a reaction with Lawesson’s reagent [206], to give an intermediate **45b** that was subsequently treated with sodium hydroxide. The desired compounds led to the release of H_2_S due to the cleavage of a phenolic ester by esterase and the consequent lactonization caused by the steric repulsion of three methyl groups (Figure 22). Remarkably, HP-102 (**46**, Figure 22) a TML derivative, was shown to reduce myocardial I/R injury and presented a cardioprotective action [207]. HP-102 is at present under investigation to better identify its chemical, pharmacological, and safety profiles prior to its further progression in clinical trials.

**Figure 23:**
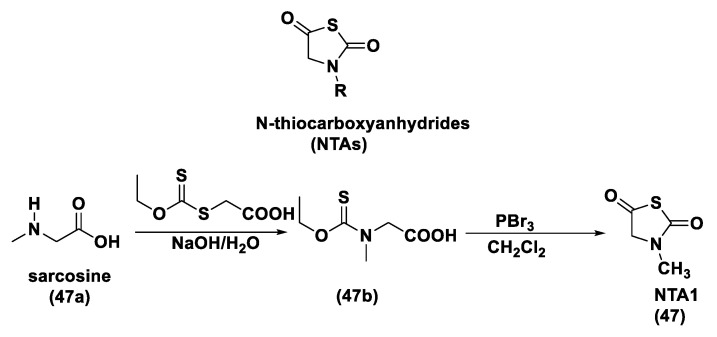
General structure of N-thiocarboxyanhydrides (NTAs) and the synthetic procedure for the synthesis of NTA1.

Carbonyl sulfide (COS)-releasing compounds are a recent class of enzyme-triggered H_2_S donors; COS represent a substrate of CA (carbonic anhydrase) enzyme that catalyzes the reaction that leads to H_2_S production [208,209]. N-thiocarboxyanhydrides (NTAs) (Figure 23) constitute dual COS/H_2_S donors [210,211], that advantageously release COS and innoxious peptide byproducts at the same time [212]. NTA1 (**47**, Figure 22), a sarcosine NTA derivative was obtained starting from sarcosine (**47a**, N-methyl glycine) as reported in Figure 23 [213].

**Figure 24:**
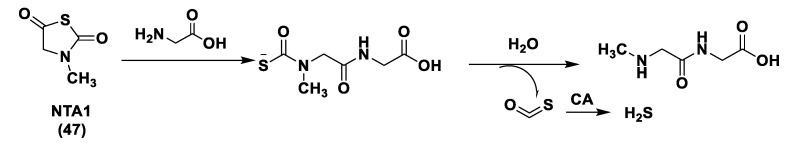
COS/H_2_S release from NTA1.

COS/H_2_S release from NTA1 is based on a cycle opening upon a nucleophilic attack promoted by endogenous amines: the dipeptide formed has been detected by LC-MS and GC-MS. The subsequent release of H_2_S from COS is due to the rapid enzymatic action of CA (Figure 24) [212]. When endothelial cells have been treated with NTA1 improved proliferation, an important step in the angiogenic process occurs [212].

**Figure 25:**
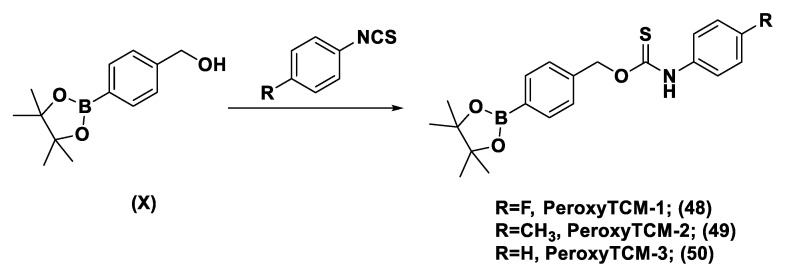
ROS-activated H_2_S donor preparation.

ROS-triggered H_2_S donors (peroxyTCM-**1**, **2**, **3**; **48**, **49**, **50**) are another class of COS/H_2_S donors; the compounds were obtained [214] by applying a ROS-cleavable aryl boronate (**X**) as a protecting group (Figure 25) [215,216].

**Figure 26:**
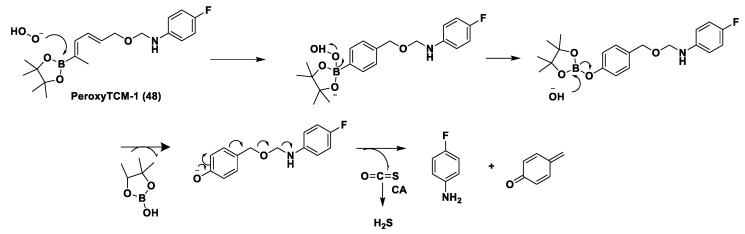
H_2_O_2_-triggered COS/H_2_S release.

The proposed mechanism for the release of COS/H_2_S is summarized in Figure 26: these compounds carry out H_2_S in the presence of an oxidant, for example H_2_O_2_: the boronate ester is transformed into a phenol by ROS, giving COS, which is then rapidly moved into H_2_S by CA [214].

Boronate esters are known as ROS scavengers and this effect was coupled with the H_2_S cytoprotective action leading to a novel tool for the treatment of human pathologies determined by elevated oxidative stress, for instance cardiovascular diseases [193]. Additional in vivo studies are ongoing for the assessment of their efficacy.

#### 3.1.5. Other H_2_S Donors in Cardiovascular Diseases

**Figure 27:**
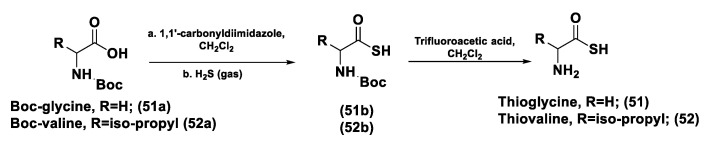
Synthesis of thioglycine and thiovaline.

Thioglycine (**51**) and thiovaline (**52**), two thioamino acids, are bicarbonate-triggered H_2_S donors [217]; these compounds have been synthesized starting from commercially accessible Boc-protected amino acids (**51a**, **52a**) that have been treated with 1,1′-carbonyldiimidazole in DCM and then with gaseous H_2_S. The subsequent deprotection of intermediates **51b** and **52b** with TFA in dichloromethane provided the required compounds (Figure 27).

**Figure 28:**
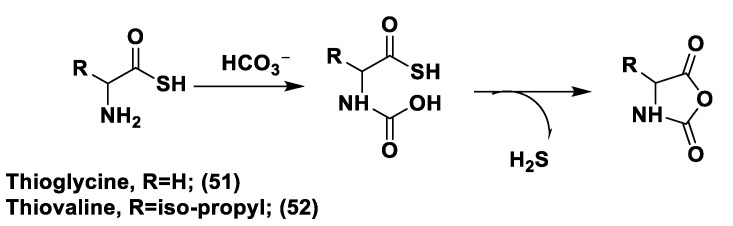
H_2_S release from thioamino acids.

Following a cyclization reaction, these compounds are converted into the corresponding amino acid N-carboxyanhydrides in the presence of a bicarbonate and produce H_2_S (Figure 28). Thioamino acids are considered as in vivo H_2_S donors due to the releasing mechanism and the high-level of bicarbonate in the blood.

Thioglycine and thiovaline H_2_S release have been assessed through amperometric studies. The compounds also improve the formation of cGMP in a concentration-dependent manner and produce a substantial relaxation of pre-contracted mouse aortic rings. Conversely, thioglycine and thiovaline are characterized by a high reactivity; in fact, they can provide amidation [218,219,220,221,222] and oxidation reactions [190], giving products which could produce undesirable side-effects.

**Figure 29:**
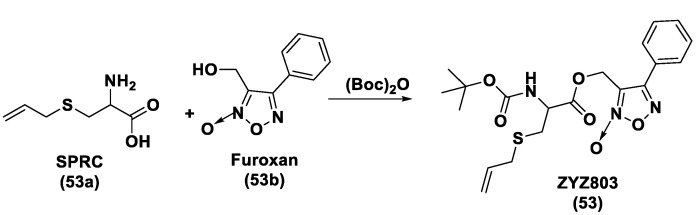
Synthesis of ZYZ803.

ZYZ803 (2-(N-Boc-amino)-3-prop-2-ynylsulfanylpropionic acid, **53**) is a novel H_2_S-NO releasing molecule, recently developed through a copulation reaction between SPRC and furoxan in the presence of Boc anhydride (Figure 29) [223,224].

**Figure 30:**
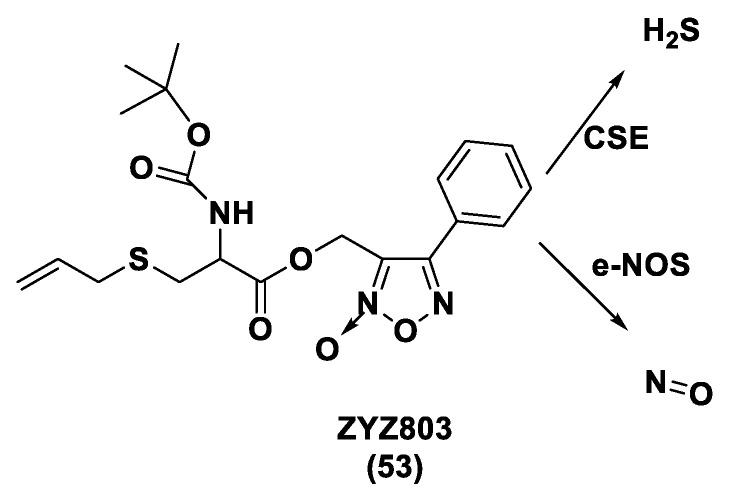
H_2_S/NO releasing pathways from ZYZ803.

SPRC (**53a**) releases H_2_S through CSE enzyme activation, whereas furoxan (**53b**) represents a thiol-triggered NO-releasing moiety [225]. Consequently, ZYZ803 (**53**) is able to stimulate either the expression of CSE through NO synthase (e-NOS) endothelial activity, releasing H_2_S and NO, correspondingly (Figure 30), promoting angiogenesis by the SIRT1/VEGF/cGMP pathway and through crosstalk between STAT3 and CaMKII [226].

In vitro and in vivo studies have shown that ZYZ803 significantly promotes endothelial cell angiogenesis [227]. In an animal model of ischemia, ZYZ803 stimulated angiogenesis in a more efficient way in comparison to the monotherapy administration of SPRC or furoxan, proving the synergistic actions of H_2_S and NO released by ZYZ803 [227]. This interesting lead compound regulated vascular tone in rat aortic rings [223], reduced cardiac dysfunction, and increased myocardial injury following heart failure [228], giving a potent cardioprotective effect.

**Figure 31:**
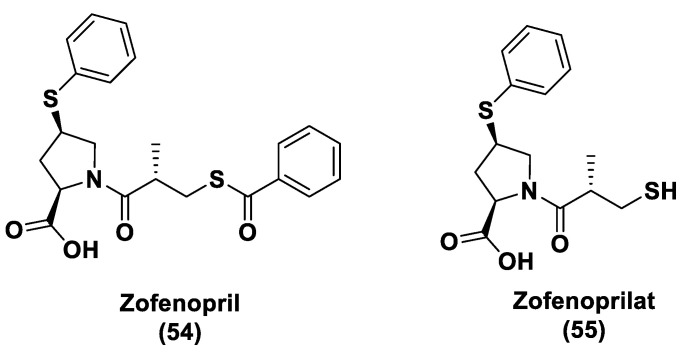
Chemical structures of Zofenopril and Zofenoprilat.

Finally, Zofenopril (**54**), an ACE inhibitor, is among the H_2_S donors with cardiovascular system activity. This compound in the liver undergoes a hydrolysis reaction with the formation of Zofenoprilat (**55**), and its active metabolite contains a thiol group (Figure 31).

Zofenopril is an already well studied drug, and it was approved for medical use in 2000. This compound has been proven to release H_2_S, and this gaseous transmitter in turn mediates the beneficial anti-apoptotic, pro-angiogenic, and anti-inflammatory effects of zofenopril [229,230,231,232,233,234]. The drug improved vascular function in a murine model of hypertension, due to its ACE inhibitor action and H_2_S-releasing effects, and the administration of this compound increased the plasmatic levels of H_2_S in mice, protecting them from myocardial I/R injury and increasing the endocardial blood flow in myocardial ischemia [235].

### 3.2. H_2_S Donors and Cancer

H_2_S has several roles in the regulation of cellular activities, for instance, migration, proliferation, apoptosis, and autophagy. A low production of H_2_S in several cancer types has been found. The principal experimental technique applied to enhance H_2_S levels in cancer cells is represented by the use of H_2_S donors; nevertheless, their use is connecting to the concentration/dose, cell type, time, and administration method.

Natural and synthesized donors have been used in this context and different effects have been observed, such as suppressant effects but also promoter effects. Following treatment with H_2_S donors, numerous signaling pathways have been altered by cells, prompting their use in cancer treatment. The good efficiency of H_2_S donors has silenced different cancer types due the regulation of cellular activities and signaling pathways. The evident opposite biological effects in different experiments, for example in the case of NaHS, needs to be further analyzed. The different effects of this salt seem to be correlated to the short-time and the high concentration of H_2_S released from NaHS, giving firstly a momentary increase in the cellular H_2_S levels, followed by a subsequent decline; this kind of release is very different to the normal slow-releasing cellular mode, and therefore NaHS has been replaced in clinical applications.

Additionally, the amount of information about H_2_S donors (such as on metabolism and clearance) is limited and their applicability has generated many questions.

For this purpose, it is important to assess the range of concentrations for H_2_S donors and to define all the potential toxicity and clearance mechanisms. Furthermore, potential interactions between different molecules, as in the case of molecular hybrids, requires additional analysis for a better realization of the side effects.

It has been observed that the combination of H_2_S donors and cancer drugs (for example cisplatin and docetaxel) increases drug sensitivity and decreases resistance. The synergistic effect of H_2_S donors with metformin/simvastatin has promoted anti-cancer activities due to the elevation of intracellular acidity and the reduction in pH regulators, paving the way for their useful administration in the treatment of advanced stages of cancer; at present, all the researchers agree that more preclinical and clinical trials are needed to study this aspect.

In summary, H_2_S donors, used both in monotherapy or in combination with other drugs, have interesting applications in cancer treatment; several studies are now in progress and the hope is to shed additional light on cancer therapy [236].

Triple-negative breast cancer (TNBC) is an aggressive subtype cancer form and represents about 15–20% of breast cancers. Unfortunately, it is characterized by metastasis and recurrence and the development of novel treatments of TNBC is very important. Recently, extraordinary progress in the treatment of TNBC by H_2_S donors has been made.

In summary, on the one hand, H_2_S donors efficiently modulate the proliferation and apoptosis of TNBC cells through the inhibition of the phosphorylation or expression of proteins connected to the NF-κB, PI3K/Akt/mTOR, and Ras/Raf/MEK/ERK signaling pathways. Otherwise, H_2_S donors inhibit the expression of MMP-2/9 and EMT to resist TNBC metastasis through the inhibition of the abnormal activation of the β-catenin pathway.

**Figure 32:**
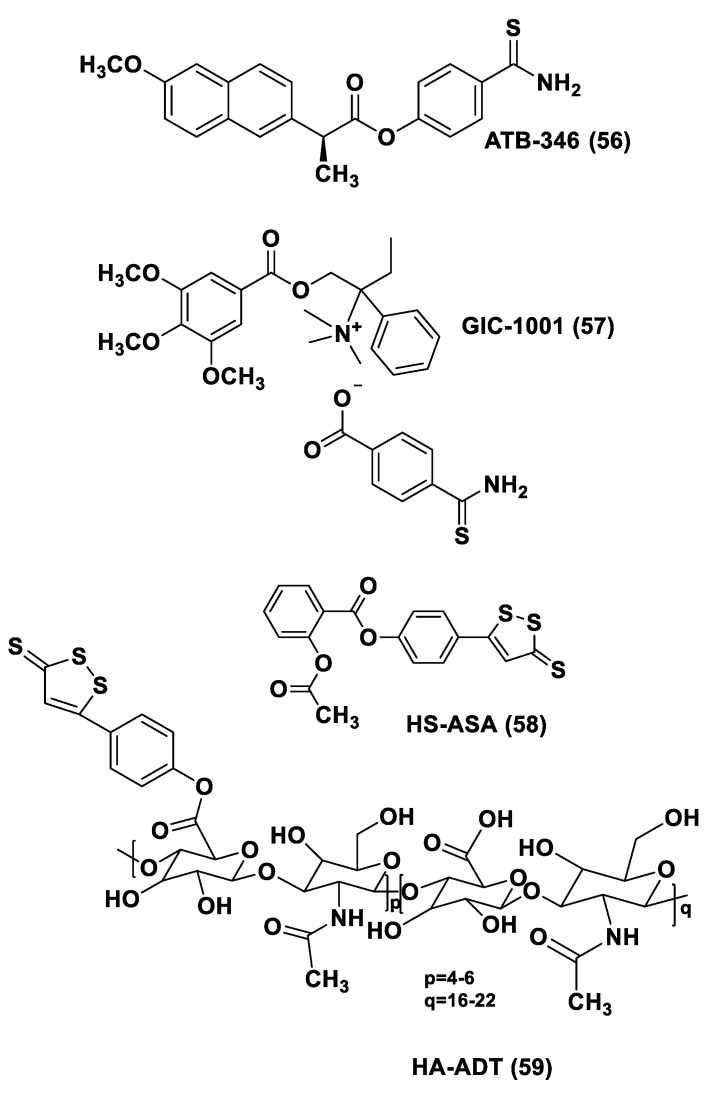
Chemical structures of H_2_S donors evaluated for TNBC treatment.

H_2_S is believed to be involved in other pathways that account for TNBC prevention and this deserves further investigation. Some organic H_2_S donors (DATS, DADS, GYY4137, ADT-OH, thiobenzamide) are used for endogenous H_2_S production to exert antitumor effects. ATB-346 (naproxen derivative, **56**, Figure 32) and GIC-1001 (**57**, Figure 32) represent some H_2_S donor-based therapeutics entered in Phase II clinical trials. HS-ASA (**58**, Figure 32) showed antitumor activity on TNBC MDA-MB-231 cells in vitro and in vivo studies. HA-ADT (**59**, Figure 32) was able to induce MDA-MB-231 cell apoptosis by improving the ratio of Bad/Bcl-xl and Bax/Bcl-2 and the expression of cleaved caspase-3/9 and cleaved PARP [237].

Hybrids capable of releasing H_2_S or NO, or both gaseous transmitters have been developed. The hybrids can be beneficial in reducing the doses of the chemotherapeutic agent in order to diminish the toxic effects of these drugs and the gaseous transmitter gains additional chemotherapeutic effects.

**Figure 33:**
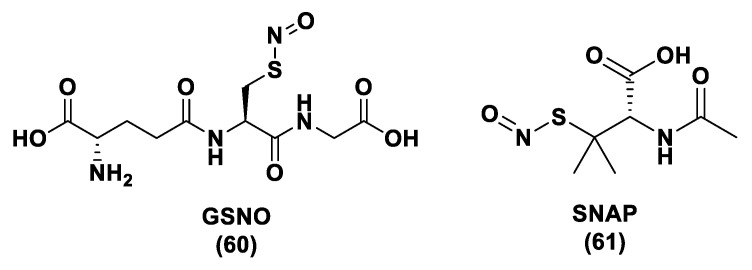
Chemical structures of GSNO and SNAP.

Various H_2_S and NO-releasing compounds have been developed for their use in cancer chemotherapy and SNOs (S-nitrosothiols) donors are to date considered the most efficient compounds. GSNO (S-nitrosoglutathione, **60**) and S-nitroso-N-acetylpenicillamine (SNAP, **61**) (Figure 33) are two SNOs that have been investigated for their anti-carcinogenic properties.

The reaction between SNO donors and Cysteine leads to the formation of CysNO at the external face of the plasma membrane. This compound has a membrane that is impermeable but passes into the cell using the L-type amino acid transporter on the plasma membrane. The reaction between SNO donors and H_2_S yields HSNO which diffuses spontaneously across cell membranes. The mixed effects of CysNO and HSNO enhance the cellular nitrosation most likely via transnitrosation processes or by enhancing the nitrosating capabilities of both compounds. Prolonged exposure of tumor cells to these compounds causes stress conditions and consequently leads to apoptosis [238].

**Figure 34:**
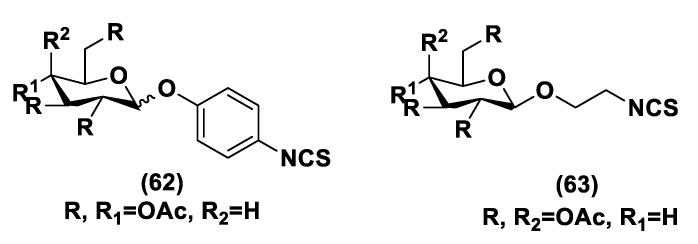
Glycoconjugated H_2_S donors.

Cancer cells are distinguished by an improved uptake of sugars, for example glucose. Fortunato et al. synthesized novel glycoconjugated-H_2_S donors in order to selectively enhance the concentration of H_2_S in cancer cells. Dithiolethione or isothiocyanate moieties have been considered for their H_2_S-releasing properties. C1-glycoconjugates have been obtained by applying a synthetic stereoselective procedure based on trichloroacetimidate glycosyl donors, while C6-glycoconjugates have been achieved through a Mitsunobu reaction (Figure 33). Subsequently the obtained compounds have been evaluated for their anticancer effects in the AsPC-1 cell line. Compounds **62** and **63** (Figure 34) have been demonstrated to release H_2_S inside the AsPC-1 cells and to modify the basal cell cycle [239].

Brassicaceae are a well-known plant family and have attracted attention for the presence of the glucosinolate myrosinase system that leads to isothiocyanates upon plant biotic and abiotic lesions.

Erucin (ERU; Figure 4), an isothiocyanate present in arugula, originates through glucosinolate glucoerucin hydrolysis due to the myrosinase enzyme.

Natural isothiocyanates modify cancer cells stimulating anti-proliferative effects at high concentrations. Gaseous hydrogen sulfide or non-natural H_2_S-releasing compounds showed comparable anti-proliferative effects.

Isothiocyanates release H_2_S in biological conditions: ERU belongs to this category and was evaluated for its anticancer effects on pancreatic adenocarcinoma cells (AsPC-1). The obtained results showed the chemo preventive and anticancer features of this compound. In addition, ERU presented significant anti-proliferative effects by the inhibition of AsPC-1 cell viability and cell migration, modifying the AsPC-1 cell cycle, and exhibiting a pro-apoptotic effect.

Moreover, AsPC-1 cells present a genetic mutation in KRAS that leads to KRAS hyper-activation and then to MAP-kinase hyper-phosphorylation. ERU repressed ERK1/2 phosphorylation. This inhibition is very important in pancreatic cancer proliferation, growth, and survival [240].

### 3.3. H_2_S Donors and Nervous System Diseases

H_2_S donors have been investigated for their use in the treatment of nervous system pathologies. The pioneer compound for this use was ACS 84 (Figure 12), a DOPA-DTT that has been shown to have an important role in Parkinson’s Disease; successively, several other compounds have been reported for their in vitro effects and for their potential use in Alzheimer’s disease (AD).

An important relationship between H_2_S and aging has been observed and a significant decline in H_2_S levels has been seen in patients affected by Alzheimer’s disease (AD). On this basis, the use of H_2_S-donors could represent an exciting and intriguing strategy to be pursued in the treatment of neurodegenerative diseases.

Sestito et al. designed a small series of multitarget molecules combining the rivastigmine-scaffold, a well-established drug already approved for AD, with sulforaphane (SFN) and erucin (ERN), two natural products derived from the enzymatic hydrolysis of glucosinolates contained in broccoli and rocket, respectively, endowed with antioxidant and neuroprotective effects.

**Figure 35:**
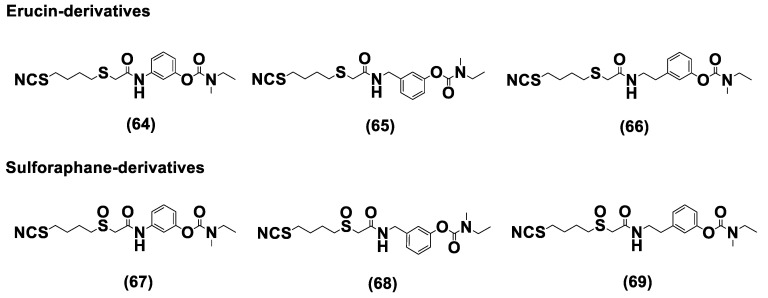
Chemical structures of ERN- and SFN-rivastigmine hybrids.

The newly obtained hybrids (**64**–**69**, Figure 35) elicited protective effects in an LPS-induced microglia inflammation model. A decrease in NO production was observed in LPS-stimulated cells pre-treated with the compounds and they also showed neuroprotective and antioxidant activities in human neuronal cells. Compound **64** proved to be the most promising agent, combining antioxidant and anti-inflammatory properties. This promising pharmacological profile has been imputed to the slow H_2_S releasing profile which combined produced a very favorable logP value [241].

**Figure 36:**
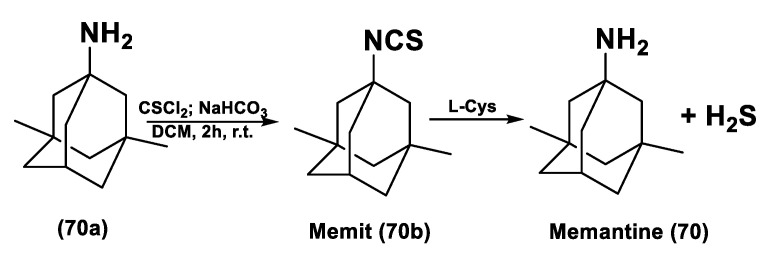
Preparation of Memit and its mechanism of H_2_S release.

In another study, Sestito et al. substituted the free amine group of memantine (**70**) with an isothiocyanate group to achieve H_2_S release. The new chemical entity, named “Memit” (**70b**, Figure 36), has been tested in vitro to establish the pharmacological profile of the “native drug”. Indeed, it has been observed that memit releases H_2_S through a cysteine-mediated mechanism, producing memantine (Figure 36). The new compound exerted protective effects in neuronal inflammation and induced a drastic decrease in ROS production. Memit has also been proven to decrease the aggregation of Aβ(1–42) and had a cytoprotective effect versus Aβ oligomers which induced damage in human neurons and rat microglia cells. Furthermore, the new molecule promoted autophagy that is altered in neurodegenerative diseases.

Memit can be considered a prodrug of memantine, although in vivo studies are important to study the substantial effects of memantine and H_2_S [242].

### 3.4. H_2_S Donors and Gastrointestinal Diseases

**Figure 37:**
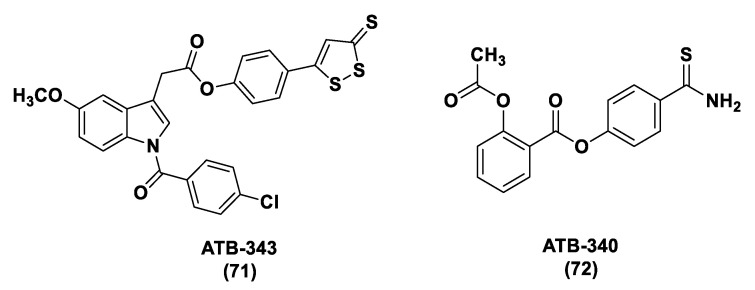
Chemical structures of ATB-343 and ATB-340.

S-aspirin (ACS-14), S-diclofenac (ACS-15), ATB-429 (Figure 13) and ATB-346 (Figure 32) have been already discussed above. In the same way, ATB-343 (indomethacin derivative, **71**) and ATB-340 (**72**) (Figure 37) decreases the gravity of NSAID-induced damage in the rat stomach. ACS-15 (CTGPharma) and ATB-337 (Antibe Therapeutics) (Figure 13), a diclofenac derivative, exhibits increased anti-inflammatory effects in comparison to the parent drug. Indeed, whereas diclofenac damages the stomach in dose-dependent manner, ACS-15 shows substantially decreased GI toxicity and does not affect hematocrit. This compound inhibits COX-1 and COX-2 activity as well as diclofenac. Moreover, differently from diclofenac, it does not cause leukocyte adherence, and is more effective in decreasing paw edema [12].

Looking in more detail at the different drug applications, ATB-429 is a mesalamine derivative and is therefore aimed at the improvement of the treatment of inflammatory bowel disease (IBD). IBD can be separated into two subtypes: Crohn’s disease (CD) and ulcerative colitis (UC). ATB-429 has been widely characterized in CD and UC animal models and is significantly superior to mesalamine. It was more efficient in treating the visceral pain associated to IBD [12]. ATB-346 represents a naproxen H_2_S donor-derivative designed for osteoarthritis pain relief. Naproxen is generally associated with a considerable risk of severe gastrointestinal bleeding and cardiovascular effects. ATB-346 showed superiority and a remarkable reduction in the gastrointestinal and cardiovascular toxicities in comparison to its parent drug in preclinical studies [12].

4-carbamothioyl phenyl 2-acetoxybenzoate (ATB-340 (**72**), Figure 37) is an H_2_S releasing aspirin. The H_2_S pathway is involved in the susceptibility of the gastric mucosa to High Fructose Diet (HFD) injury. The administration of ATB-340 in elderly patients showed an increased efficiency in the decrease in age-associated gastric mucosal defense. Decreased activation of H_2_S signaling in the HFD model is associated with the increase in mitochondrial impairment and oxidative stress that stimulates the expression of thiosulfate-dithiol sulfurtransferase. The gastro-protection observed upon ATB-340 treatment is H_2_S-dependent and is related to the attenuation of oxidative stress [243].

### 3.5. H_2_S Donors and Dermatological Diseases

In the skin, H_2_S is generated by enzymic pathways and has a physiological role in various functions, as well as inflammation, vasodilatation, cell proliferation, and apoptosis. Modifications of H_2_S production are involved in several dermatological diseases, for example melanoma, psoriasis, and other dermatoses.

The data available to date in relation to H_2_S signaling in the skin support the involvement of hydrogen sulfide in patho-physiological processes. Generally, H_2_S effects are highly concentration or dose-dependent, implying the interaction with targets that result in stimulatory or inhibitory effects that interfere with skin processes.

Due to the important role of H_2_S in skin physiology, altered H_2_S production contributes to the pathogenesis of various cutaneous diseases.

**Figure 38:**
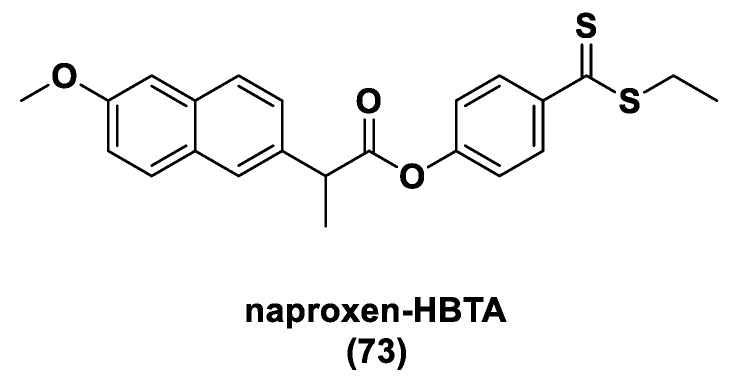
Chemical structure of naproxen-HBTA.

Several H_2_S-releasing compounds, for example Na_2_S, NaHS, DATS (Figure 4), GYY-4137 (Figure 6), JK1 (Figure 8), ATB-346 (Figure 32), and naproxen-HBTA (naproxen-4-hydroxybenzodithioate, **73**) (Figure 38), have been examined in experimental studies.

These compounds are potential therapeutic molecules useful for the treatment of several skin diseases due to their different etiological aspects. The development of novel and safer H_2_S-based drugs will fundamentally depend on increasing knowledge of the exact role of H_2_S in the various aspects of skin biology [100].

In 2019, naproxen-HBTA, a H_2_S-releasing derivative of naproxen, was analyzed in a metastatic melanoma model in in vitro and in vivo studies. The obtained results demonstrated that naproxen-HBTA stimulated caspase 3-mediated apoptosis and reduced motility and invasiveness. Moreover, daily oral treatment with naproxen-HBTA significantly suppressed melanoma growth and progression in mice. This dual approach could represent a “combination therapy” for melanoma based on COX-2 and H_2_S pathways as new therapeutic targets [244].

### 3.6. H_2_S Donors and Viral Infections

As stated above, H_2_S exerts several positive effects in pathologies characterized by lung inflammation, such as COVID-19. H_2_S promotes the elimination of potentially dangerous viruses by breaking mucin disulfide bonds, making the mucus less viscous and facilitating the expulsion of pathogens through the respiratory ciliary apparatus.

Through S-sulfuration, H_2_S impedes the activation of the NF-κB pathway, and the inhibition of the IKκβ enzyme, inhibiting the translocation of NF-κB into the nucleus, and then the generation of the cytokine storm. Moreover, H_2_S stimulates the activation of Nrf2, improving the expression of enzymes and antioxidant molecules. Furthermore, H_2_S protected the tissues from oxidative stress due to direct antioxidant activity. Finally, H_2_S promoted bronchodilation by activating the KATP channels expressed on the cell membrane of the bronchial smooth muscle cells and leading to electrolyte absorption by increasing muco-ciliary clearance and blocking the Na^+^/K^+^ ATPase pump.

Antiviral effects of organosulfur molecules have been investigated [103] and recent studies have elucidated that these molecules are H_2_S donors, leading to the belief that antiviral effects are linked to the release of H_2_S.

DATS (Figure 3) has been tested in cytomegalovirus (CMV)-induced hepatitis. In this study, the polysulfide DATS decreased the viral load of CMV in the infected organs of mice and humans by inhibiting gene transcription and promoting the amplification of CMV-induced T-regulatory helper cells, giving an immunosuppressive effect versus the CMV noted in murine models [103].

**Figure 39:**
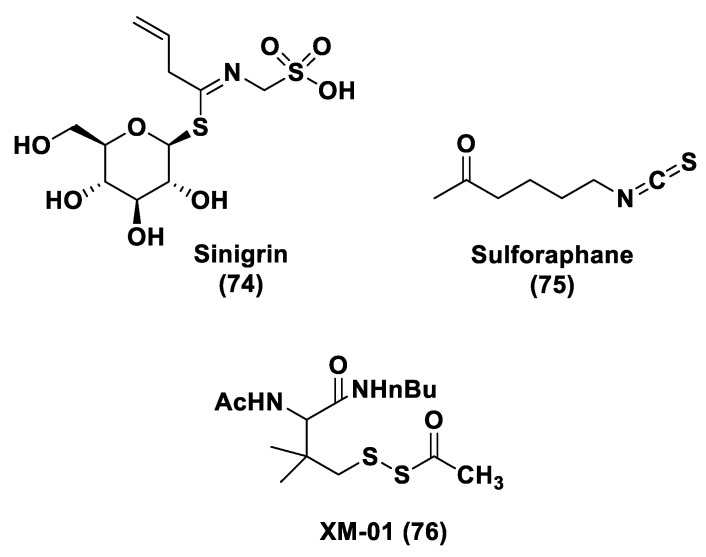
Chemical structures of sinigrin, sulforaphane, and XM-01.

Similarly, the antiviral properties of the root extract of the plant *Isatis indigotica* L. have been investigated. This extract contains sinigrin (a precursor of the allyl isothiocyanate, (**74**) Figure 39), a compound that has been proven to be the most effective compound in 3CLpro inhibition (3-chymotrypsin-like protease; Mpro—main protease) of SARS-CoV. Notably, this protease causes the proteolytic processing of polypeptides leading to functional proteins required for viral replication [103].

Data obtained exploring the effects of sulforaphane (**75**, present in various plants of Brassicaceae family; Figure 39) suggest that the antiviral effect of Brassicaceae extracts can be largely ascribed to their isothiocyanate compounds. Sulforaphane was proven to have antiviral activity through the influenza A/WSN/33 (H1N1) virus by decreasing virus replication in vitro. These results have been confirmed by a randomized, double-blind, placebo-controlled clinical study. Sulforaphane improved virus-induced granzyme B (a protease released by NK or T cells that stimulates cell apoptosis) through the NK cells. The increase in granzyme B levels was adversely related to influenza RNA levels in nasal lavage fluid cells, proving a sulforaphane antiviral role [103].

GYY4137 (Figure 3) has been used by the Casola group for studying the antiviral role of H_2_S in RSV (respiratory syncytial virus). RSV infection is characterized by low levels of CSE mRNA and protein expression in airway epithelial cells, consequently leading to a reduction in the levels of H_2_S. Scientists have reported increased viral replication and airway inflammation in RSV-infected CSE knockout mice in comparison to wild-type mice. Bearing in mind these findings, GYY4137 has been used in order to set the role of H_2_S in paramyxovirus infections and a considerable reduction in the production and viral replication of pro-inflammatory mediators was found. The antiviral effect of this compound was shown to be related to the inhibition of syncytia formation, viral assembly, and release. Moreover, GYY4137 revealed anti-inflammatory effects by the regulation of NF-κB and IRF-3 activation. This effect was confirmed in vivo during RSV infections through the intranasal administration of GYY4137 to infected mice: the compound inhibited viral replication when administered within 24 h of infection, decreased pro-inflammatory mediators, and reduced airway dysfunction. These effects have also been observed in vitro even on different RNA virus infections, for example influenza A and B and Ebola virus.

In in vitro and in vivo models, *gem*-dithiol-based H_2_S donors reduced viral replication and deceased proinflammatory mediators, confirming once again that H_2_S was responsible for the observed antiviral effects [103].

More recently, XM-01 (**76**; Figure 39) a disulfide compound, has been investigated for its antiviral effect on different viral strains (RSV, influenza virus (A/WSN/33 strain), rotavirus). The effects of XM-01 (a cysteine-based perthiol derivative) correlated with its H_2_S release, were reported in a study based on the identification of cardioprotective H_2_S donors. Moreover, XM-01 demonstrated antiviral effects on enveloped viruses but did not show activity through non-enveloped viruses.

Thus, as the antiviral action may be due to alterations of the viral membrane, GYY4137 and its analogues could be useful against enveloped viruses inhibiting the entry phase of the host cell.

In a very recent paper, H_2_S antiviral activity versus SARS-CoV-2 has been reported describing its interference with ACE and TMPRSS2 [103].

### 3.7. H_2_S Donors and Other Diseases

H_2_S is implicated in other diseases, such as diabetes, pain, and bone tissue alterations.

Type 2 diabetes mellitus (DM) represents an important chronic disease correlated to severe vascular and microvascular complications (including neuropathy, cerebrovascular and peripheral arterial diseases, and retinopathy), that often leads to the progression of atherosclerosis.

Dietary therapy represents the first step in the treatment of diabetic patients. Medicinal plants are growing in importance among the current therapeutic options due to their effective and economical therapeutic support and reduced side effects.

Organosulfur compounds present anti-inflammatory, antioxidant, hypoglycemic, and immunomodulatory effects; for these effects they have usually been considered as cardioprotective agents in type 2 DM and therefore, organosulfur compounds derived from garlic (Figure 4), signify an appreciated support in the type 2 DM diet.

Several studies, ranging from preclinical to clinical studies, have demonstrated the relevant effects of hydrogen sulfide in human DM; however, further evidence is necessary to establish the validity of the administration of organosulfur compounds in the treatment of DM. Globally, stability is one of the most relevant problems with natural compounds; in fact, allicin and its derivatives (Figure 4), quickly degrade. Consequently, to improve stability and bioavailability, several researchers are developing new strategies for the administration of these natural compounds (nano-emulsions, capsules containing garlic oil). The preparation of combined formulations containing other nutraceuticals, with synergistic effects, is a very focused challenge for many companies. The variability of the chemical composition of vegetables, dependent on environmental conditions and countries of production, represents another relevant problem. Therefore, vegetable trials have to contain information on the chemical composition and standardized preparations. The study of garlic organosulfur compounds represents a stimulating field where inflammation, redox biology, tissue repair, detoxification, and regeneration are connected for favorable effects on human health [245].

Trimebutine maleate, a spasmolytic FDA and EMA-approved drug, has been recently evaluated for the treatment of patients undergoing sedation-free colonoscopy. A new trimebutine H_2_S-releasing salt has been recently disclosed and has been proven to release in vivo the gasotransmitter that allows a reduction in nociception. The new compound (GIC-1001 (**57**), Figure 32) has been obtained combining trimebutine and 3-thiocarbamoylbenzoate (3TCB). It has been tested in a mouse model of colorectal distension: the oral administration of GIC-1001 led to a significant reduction in the nociceptive responses to all noxious stimuli. This effect was significantly superior in comparison to the parent compound at equimolar doses. The preliminary results of GIC-1001 suggest that this compound may represent a good compound to reduce the visceral pain and discomfort caused by lumenal distension [246].

**Figure 40:**
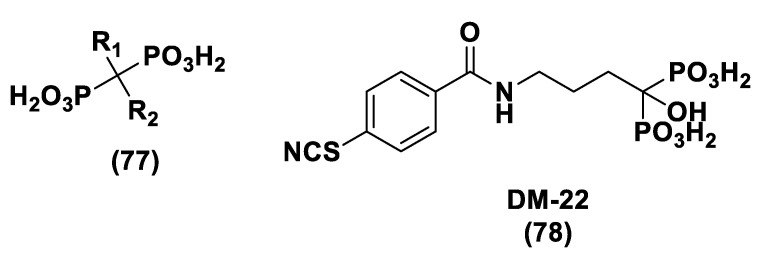
General chemical structure of BPs and chemical structure of DM-22.

BPs (Bisphosphonates, **77**, Figure 40) represent the principal treatment for bone loss due to various pathological conditions. Considering their high bone affinity, BPs have been utilized to develop conjugates with anti-catabolic and pro-anabolic drugs. H_2_S stimulates osteogenesis and reduces osteoclast differentiation; following this rationale, an innovative compound, DM-22 (**78**, Figure 40), has been prepared combining alendronate (AL) and an arylisothiocyanate. DM-22 and AL have been tested in vitro to evaluate the effects on h-OCs (human osteoclasts) and h-MSCs (mesenchymal stromal cells) during osteogenic differentiation. Amperometric analysis showed that DM-22 slowly produces H_2_S in a thiol-dependent manner. DM-22 significantly reduced h-OCs differentiation and function, preserving a residual viability of h-OCs. In h-MSCs, DM-22 did not produce cytotoxicity as showed by a LDH assay, considerably stimulating bone mineralization, and enhancing mRNA expression of Collagen I.

DM-22 is therefore a new bisphosphonate without cytotoxicity and represents a candidate for a different kind of osteoanabolic drug [247].

### 3.8. Other H_2_S Donors

In this section further examples of H_2_S donors recently reported in the literature are discussed.

**Figure 41:**
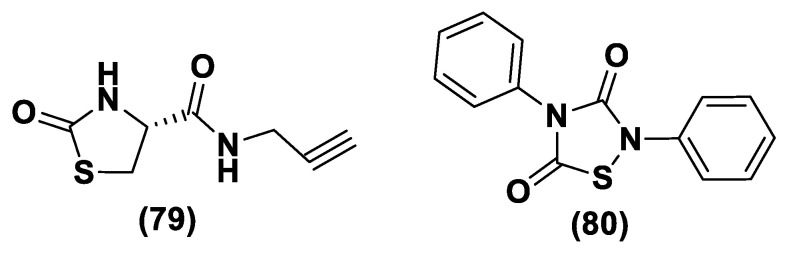
Chemical structures of compound **79** and **80** in [248,249].

Research in the field of H_2_S is at present compromised by the lack of pharmacological tools, for example, selective enzymatic inhibitors exclusively target only one of the H_2_S pathway components. In 2016, Corvino et al. applied a new approach based on a hybrid method to design a novel selective CSE inhibitor. A library of compounds was developed; oxothiazolidine derivatives (**79**, Figure 41) demonstrated maximal inhibitory effects in tissues and were shown to be superior to PAG (propargylglycine). Moreover, metabolomics assays on the purified enzymes demonstrated their full selectivity as CSE inhibitors. In comparison to PAG, compound 79 behaved in a different way: both compounds inhibited CSE through the production of pyruvate, ammonia, and H_2_S, but unexpectedly, in the presence of PAG, CSE used the substrate L-Cys and produced cystine and lanthionine. In contrast, 79 completely inhibited the enzyme, without an effect on the concentration of the substrate. This resulted in the chance of utilizing lower doses of 79 in comparison to PAG, thus decreasing all the adverse effects. Compound 79, but not PAG, is not a suicidal inhibitor and this represents an additional advantage. In order to further progress into clinical studies, a careful pharmacological investigation will be required in order to evaluate whether the new compound is associated with unwanted side effects. This highly selective CSE inhibitor could be useful to better understand the role of CSE vs. CBS in the pathophysiology of the diseases in which a role for the H_2_S pathway has been suggested. The development of selective CSE inhibitors, particularly characterized by good water solubility, will further shed new light on the crosstalk between H_2_S and other relevant pathways of transmitters (e.g., NO, CO) [248].

More recently, Severino et al. reported the preparation of novel H_2_S donors bearing an 1,2,4-thiadiazolidine-3,5-diones scaffold as new donors for a controlled release of H_2_S. Amperometric approaches have been used to evaluate the H_2_S-releasing properties and the vasorelaxant ability on aorta rings of the new compounds. Moreover, a study on the release mechanism has been conducted on THIA 3 (**80**, Figure 41), the most efficient H_2_S donor; the modification of structural substituents determined a reduction in the release of H_2_S. The influence of THIAs on the vasoconstrictor effects of PE (L-phenylephrine) was tested on isolated mouse aortic rings, proving that the presence of THIA 3 completely reverted the PE effect in evoking vasoconstriction. By using HPLC, MS, and NMR techniques, the molecular mechanism responsible for H_2_S release has been studied. 1,2,4-thiadiazolidine-3,5-dione has been confirmed as a H_2_S-releasing moiety and its H_2_S releasing properties have been modulated by substitutions on the aromatic rings [249].

In 2016 Marino et al. reported 4-carboxy-phenyl isothiocyanate as a new H_2_S donor.

As reported above, H_2_S regulates inflammation and allergic reactions, in which mast cells represent major effector cells. IgE receptor (FcεRI) cross linking gives an enhancement to intracellular calcium ([Ca^2+^]i), a crucial step in mast cell degranulation. The role of H_2_S in a [Ca^2+^]i-dependent mast cell activation has been evaluated and the effects of H_2_S, principally released from PhNCS-COOH (Figure 21), on the degranulation of native murine mast cells has been analyzed, as well as its effect on RBL-2H3 mast cell lines. The release of β-hexosaminidase and renin, two specific mast cell degranulation markers, has been described, together with changes in [Ca^2+^]i and the phosphorylation of proteins downstream of FcεRI activation. H_2_S released by PhNCS-COOH decreased degranulation and renin release in all mast cell types. Moreover, PhNCS-COOH also inhibited the increase in [Ca^2+^]i caused by Ca^2+^ ionophore, FcεRI activation, and thapsigargin. Additionally, PhNCS-COOH diminished the phosphorylation of Syk, cPLA-2, and PLCγ1 in antigen-stimulated RBL-2H3 cells. Taken together, the results demonstrated that H_2_S reduces [Ca^2+^]i availability through the reduction in the phosphorylation of proteins downstream of FcεRI cross-linking on the mast cells, and then mast cell degranulation and renin release. The data indicate that PhNCS-COOH is a therapeutic instrument in mast cell-mediated allergic disorders [250].

**Figure 42:**
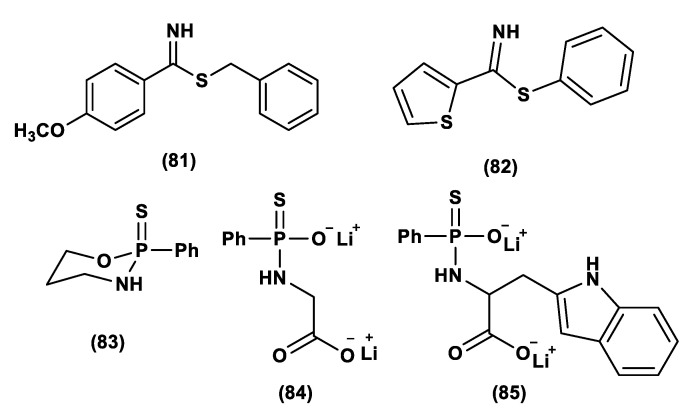
Chemical structures of compounds **81** and **82** in [251] and compounds **83**, **84,** and **85** in [252].

In 2017, a new set of iminothioethers (ITEs) with H_2_S-releasing properties and characterized by vasorelaxing effects on rat aortic rings was reported. Compounds **81** and **82** (Figure 42), were chosen as representative of slow and fast rate H_2_S donors, correspondingly, a full recovery of the basal coronary flow, giving back the AngII-induced effects in isolated rat hearts was determined. Studies on HASMCs (human aortic smooth muscle cells) showed membrane hyperpolarizing effects, linked to the intracellular generation of H_2_S. These findings highlighted compounds 81 and 82 as new pharmacological H_2_S donors useful for cardiovascular drug discovery [251].

In 2019, two series of thiophosphamide derivatives were studied in vitro and in vivo. Compounds **83** and **85** (Figure 42) were shown to release H_2_S under measured condition; the H_2_S-releasing rates in tissues and organs were different in PBS, but the H_2_S-releasing mechanism was the same. All the thiophosphamide derivatives exhibited low toxicity when tested in five cancer cell lines and in W138 cell lines and these significantly decreased the level of TNF-α and improved the level of IL-10. Moreover, the compounds preserved H9c2 cells from injury and myocardial injury by an antioxidation effect. Compounds **84** and **85** (Figure 42) proved to be the most promising derivatives. In conclusion, these thiophosphamide derivatives showed low toxicity, great activity, and a good pharmacokinetic profile and could be candidates for additional investigation [252].

Recently, thioamides have attracted attention especially when included with H_2_S–NSAID hybrids and showed low toxicity, but regrettably the mechanism of H_2_S release from thioamides continues to be unclear.

**Figure 43:**
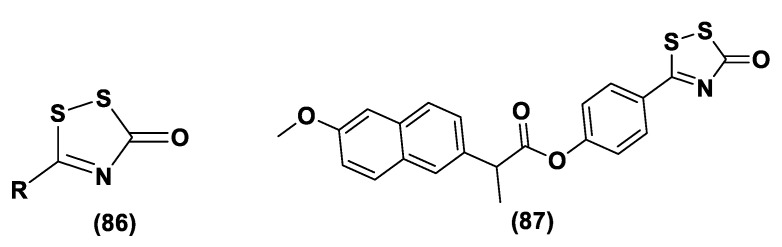
General chemical structure of **86** and the chemical structure of **87** in [253].

The synthesis and the evaluation of SulfenylTCMs, (**86**; Figure 43), a H_2_S-releasing class of thioamide-derived sulfenyl thiocarbamates, has been described to overcome this issue. SulfenylTCMs release COS (carbonyl sulfide), which is immediately converted into H_2_S by CA (carbonic anhydrase), due to the activation by cellular thiols. These compounds showed excellent H_2_S release and worked by mechanistically well-defined pathways. The sulfenyl thiocarbamate group is easily bonded to common NSAIDs, for example naproxen, to generate new hybrids, such as YZ-597 (**87**, Figure 43), able to release H_2_S in cellular environments. This new class of H_2_S donors offers an important starting point for the development of new donors [253].

In 2019, Levinn et al. reported a library of first-generation COS/H_2_S donors based on self-immolative thiocarbamates. By using specific triggers and activators such as biological thiols and bio-orthogonal reactants, the release of COS was shown. Moreover, a series of H_2_O_2_/reactive oxygen species (ROS)-triggered donors were reported and the probable mechanism of activation of these compounds caused by endogenous levels of ROS production was shown. Through orthogonal activation approaches, the scientists proved that o-nitrobenzyl photocage-functionalized donors facilitated access to light-activated donors. Moreover, Levinn et al. reported a cysteine-selective COS donor activated by a Strongin ligation mechanism and a simple esterase-activated donor, with fast COS-releasing kinetics and a capability to inhibit mitochondrial respiration in BEAS-2B cells. Further experiments showed that COS release rates and cytotoxicity were directly linked to these compounds. Finally, more recently, donors capable of generating a colorimetric or fluorescent optical response due to COS release have been reported [254].

**Figure 44:**
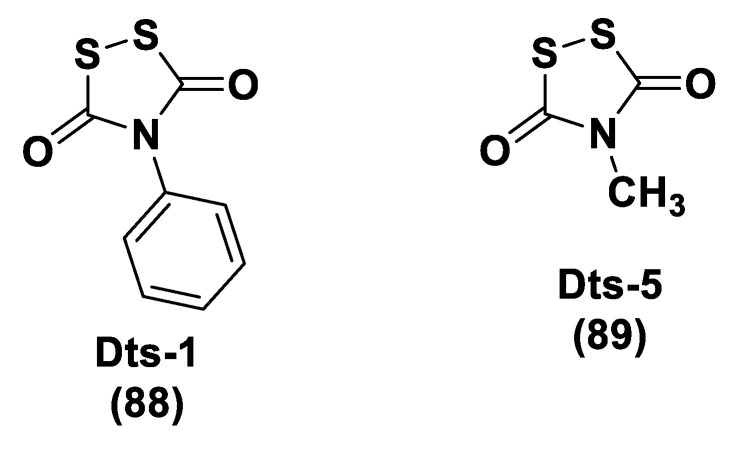
Chemical structures of Dts-1 and Dts-5 reported in [255].

In 2020, Zhou et al. reported N-dithia-succinoyl amines as thiol-triggered COS/H_2_S donors. Indeed, GSH, homocysteine and other thiols prime the release of COS and H_2_S from these compounds by an unexpected mechanism. Significantly, Dts-1 and Dts-5 (**88**, **89**; Figure 44) represent two analogues that showed intracellular H_2_S release, and Dts-1 imparted important anti-inflammatory effects in LPS-challenged microglia cells [255].

**Figure 45:**
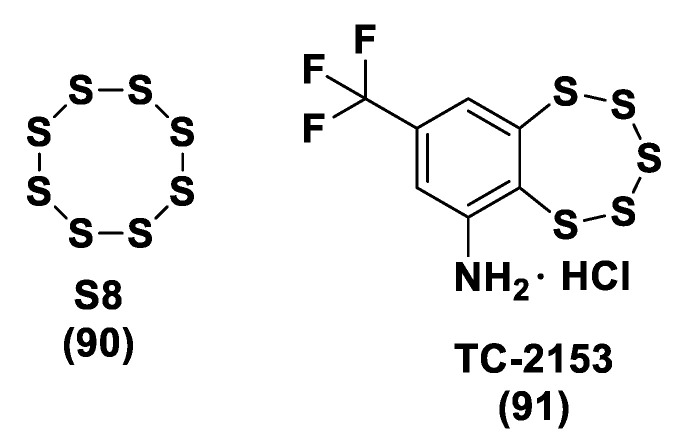
Chemical structures of S8 and TC-2153.

The scientific community is oriented towards the design of polymer systems for the release of H_2_S as therapeutics. Supramolecular tuning of the properties of compounds characterized by chains of sulfur atoms (DATS—Figure 3; S8 (**90**) Figure 45) has been shown to give superior donors, for example DATS-loaded polymeric nanoparticles.

SG1002 is a water-insoluble, alpha-sulfur rich microcrystalline material including traces of ionic substances (sodium sulfate, sodium thiosulfate, and sodium polythionates). Promising results in animal models of several diseases (for example heart failure, ischemic damage, atherosclerosis, Duchenne muscular dystrophy) have been found by using this compound. Compound **91** (TC-2153, 8-(Trifluoromethyl)-1,2,3,4,5-benzopentathiepin-6-amine hydrochloride, Figure 45) gave good results in age-related memory decline, Alzheimer’s disease, fragile X syndrome, schizophrenia, and cocaine addiction. Finally, DATS has been tested in colon, gastric, brain, and breast cancer. SG1002 could represent the preferred H_2_S donor for the evaluation of the gasotransmitter’s effects in animal models, due to its distinctive capability to slowly produce H_2_S without by-products and in a dose-independent and enzyme-independent sustained mode [256,257].

**Figure 46:**
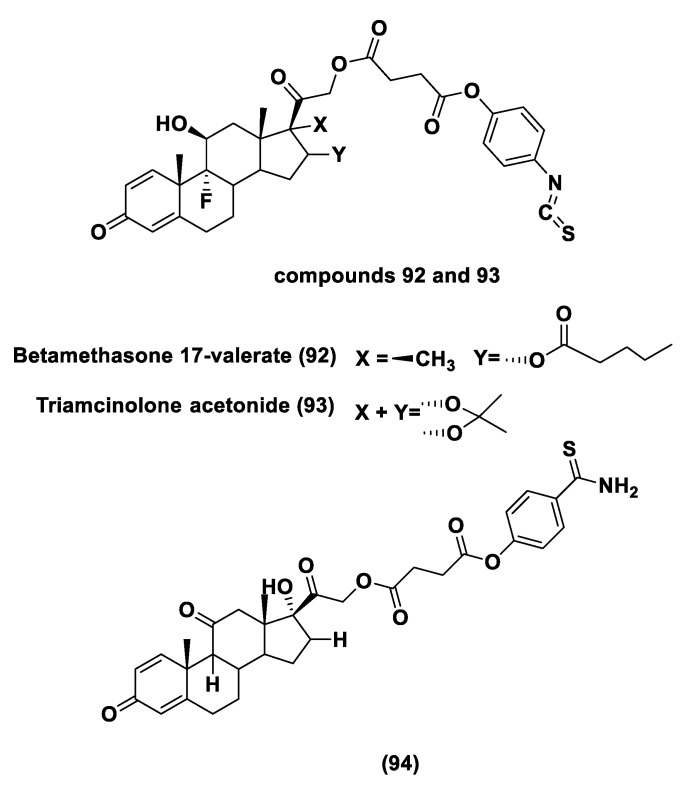
Chemical structures of compounds **92** and **93** in [258] and compound **94** in [259].

In a very recent paper, Giordano et al. reported hybrids between H_2_S-donors and betamethasone 17-valerate or triamcinolone acetonide. Considering the broad use of glucocorticoids for the treatment of inflammation in asthmatic patients and the positive effect of H_2_S on this disease, novel betamethasone 17-valerate and triamcinolone acetonide hybrids linked to H_2_S-donor moieties have been designed and synthesized. The obtained compounds were assessed for the potential H_2_S-releasing profile in a cell-free environment and in BSMCs (bronchial smooth muscle cells). Molecular modelling studies carried out with two hybrids **92** and **93** (Figure 46) have been reported and the results showed that their different H_2_S-releasing properties may be due to the steric accessibility of the isothiocyanate carbon atom. Moreover, the most interesting hybrids **92** and **93** have been examined for their inhibitory effect on mast cell degranulation and for their capability to produce cell membrane hyperpolarization in BSMCs. Important inhibitory effects on mast cell degranulation have been observed, due to a reduction in the release of β-hexosaminidase which was more effective compared to the corresponding native drugs. Compounds **92** and **93** established an important membrane hyperpolarization of BSMCs and have been shown to be more effective than the reference compound NS1619 [258].

In a similar paper, Corvino et al. reported a library of hybrids obtained by the condensation of corticosteroids with different H_2_S donor moieties. Through an amperometric study, all the hybrids released H_2_S and their chemical stability was explored. The prednisone–TBZ hybrid (**94**, Figure 46) was chosen for additional evaluations. The results of in vitro and in vivo studies evidently showed the anti-inflammatory properties of the released H_2_S. Due to the protective effect on airway remodeling, compound **94** can be considered an interesting molecule for reducing allergic asthma symptoms and exacerbations [259].

## 4. Conclusions

Considering the therapeutic potential of H_2_S, H_2_S donor chemistry has remarkably progressed. The progress of chemical synthesis has acted as the driving force for H_2_S research, and has particularly focused on the preparation of H_2_S donors useful for biological studies on the physiological and pathophysiological effects of this molecule.

Some H_2_S donors are or have been under pharmacological evaluation in clinical trials. Remarkable examples include GIC-1001 (GIcare Pharma Inc.), a trimebutine maleate salt and a H_2_S donor used as an alternative to sedatives in colonoscopies; ATB-346, a naproxen-based H_2_S donor (Antibe Therapeutics), that shows anti-inflammatory effects and that, in 2016, was included in a phase II study concerning the pain associated with osteoarthritis; SG-1002 (SulfaGENIX), that has been assessed as part of an investigation aimed at increasing circulating H_2_S and NO levels subsequently heart failure; ammonium tetrathiomolybdate (ATTM, (NH_4_)_2_MoS_4_), which has been subjected to clinical trials for the treatment of breast cancer owing to its copper depletion effects; and finally, human clinical studies have supported the chemo preventive effects of sulforaphane on carcinogenesis [260] and antiviral action through the influenza A/WSN/33 (H1N1) virus.

Crucial doubts persist in this field and these issues will necessitate combined efforts from chemists, biologists, and pharmacologists. One question is related to the determination of the H_2_S therapeutic window in different targets; another is the elucidation of the pathways responsible for donor effects. Clarification of the biological activity and the species involved will be necessary to increase the biological knowledge of donor triggers and by-products, species interchange, signaling mechanisms, and redox chemistry of H_2_S donors. Moreover, the release of reactive by-products, including unidentified sulfur species, from H_2_S donors represent a topic that needs further attention.

All these aspects must necessarily be considered in order to broaden this field. Incessant innovation in synthetic donors and an improved knowledge of H_2_S physiology are paving the way for H_2_S therapeutics to be used in clinics.

## Data Availability

No new data were created or analyzed in this paper. Data sharing is not applicable to this article.
